# Clinical Effectiveness of Immersive Virtual Reality Exercise Interventions: Systematic Review and Meta-Analysis of Randomized Controlled Trials

**DOI:** 10.2196/87542

**Published:** 2026-04-20

**Authors:** Riley C C Brown, Megan H Ross, Trevor G Russell

**Affiliations:** 1 RECOVER Injury Research Centre, The University of Queensland, Level 7, Surgical Treatment and Rehabilitation Services (STARS), Herston, Queensland, 4029, Australia, 61 33655560

**Keywords:** virtual reality, immersive virtual reality, physical activity, effectiveness, feasibility, exercise

## Abstract

**Background:**

Physical inactivity remains a global health concern, with only one in 5 adults meeting combined aerobic and muscle-strengthening guidelines. Exercise interventions delivered through immersive virtual reality (IVR) offer a novel mode of delivery. Little is known about the clinical effectiveness or feasibility of exercise via IVR across population groups. A detailed understanding of clinical effectiveness and feasibility is required for clinicians to decide whether to include IVR in exercise practice.

**Objective:**

The objective of this systematic review was to assess the clinical effectiveness of IVR interventions using aerobic or anaerobic exercise.

**Methods:**

A systematic review incorporating meta-analyses was conducted. Searches were conducted across PubMed, Embase, Web of Science, and CINAHL from inception until January 6, 2026. Randomized controlled trials including participants with an acute health condition, chronic disease, history of reconstructive or restorative surgery, and older adults implementing IVR exercise and reporting clinical effectiveness outcomes were included. Random effects meta-analyses were conducted for between-group comparisons for clinical effectiveness outcomes, grouped according to comparator group activity (exercising/nonexercising). Risk of Bias was assessed using the Cochrane Risk of Bias 2 tool and the certainty of evidence with Grading of Recommendations, Assessment, Development, and Evaluation.

**Results:**

Twenty-six trials with 846 total participants were included in this review, with 23 progressing to meta-analyses. Pooled analyses revealed a general trend for IVR, but no statistical differences with comparator intervention (exercising or nonexercising) for mobility and functional balance (exercising: standardized mean difference [SMD] −0.345, 95% CI −1.095 to 0.406; *P*=.29; nonexercising SMD −0.322, 95% CI −0.931 to 0.288; *P*=.22), functional leg strength (exercising: SMD −0.161, 95% CI −0.573 to 0.250; *P*=.33; nonexercising: SMD −0.351, 95% CI −1.750 to 1.049; *P*=.48), quality of life (exercising: SMD 0.036, 95% CI −0.444 to 0.516; *P*=.84; nonexercising: SMD −0.053, 95% CI −0.839 to 0.728; *P*=.80) or other outcome domains. Eighty percent of outcomes assessed were rated as “some concerns” (n=16) or at “high” (n=21) risk of overall bias. Grading of Recommendations, Assessment, Development, and Evaluation certainty grading was deemed to be “low” or “very low” for all outcomes.

**Conclusions:**

This systematic review incorporating meta-analyses provides initial evidence for the clinical effectiveness of IVR exercise interventions. This review differs from previous literature by systematically collecting and appraising evidence exclusively from IVR aerobic/anaerobic exercise interventions from across a variety of populations and settings, and including a broad range of clinical effectiveness outcomes. Initial evidence may suggest that IVR exercise does not seem to statistically differ from comparators for clinical effectiveness outcomes. However, high heterogeneity, substantial risk of bias among trials, and “low” to “very low” certainty in evidence reduce overall confidence in the findings. While these results indicate that IVR may be a viable option for the delivery of exercise, a more robust methodology in future trials is needed to properly verify findings and improve certainty. This will help to determine the real-world applicability of IVR exercise interventions for the improvement of health-related measures.

## Introduction

Physical inactivity is a substantial public health concern directly responsible for more than 7% of all-cause and cardiovascular disease deaths globally [1]. In 2022, the global age-standardized prevalence of adults not meeting the recommended guidelines of at least 150 minutes of moderate-intensity aerobic physical activity was 31% [2], with projections forecasting an increase to 35% by 2030 [3]. When expanded to account for muscle-strengthening recommendations (at least 2 sessions per week), one in 5 adults meets the combined guidelines [4]. Exercise interventions incorporating aerobic and anaerobic (eg, muscle strengthening activities) have been shown to improve disease control and reduce cardiovascular risk for a variety of cohorts [5]. However, traditional exercise programs often have low rates of adherence, particularly in chronic disease cohorts (≈50%) [6]. This highlights a persistent implementation gap and underscores the need for novel approaches to exercise delivery to promote sustained physical activity and exercise adherence. Digital health technologies may represent an effective and accessible form of physical activity intervention in clinical cohorts [7].

Immersive virtual reality (IVR) is an advanced form of digital health and computer-human interaction that immerses users in a 3D, computer-generated environment using a head-mounted display (HMD) [8]. Early research suggests that IVR may serve as an acceptable and effective strategy for delivering cognitive and psychological interventions across multiple populations [9-14]. Contemporary research also suggests that physical rehabilitation (eg, balance and range of motion training) incorporating IVR is comparable to conventional approaches for stroke motor recovery [15-17] and musculoskeletal pain improvement [18,19]. Furthermore, a 2024 systematic review suggests that IVR can be used as a tool to support physical activity promotion and psychological well-being through reduced perceived exertion, increased enjoyment, and improved self-efficacy [20]. Although these interventions appear to positively influence physical activity and exercise adherence in different populations [20-22], the extent to which IVR translates into meaningful improvements in health-related outcomes remains uncertain.

While research is limited compared with cognitive or physical rehabilitation, IVR exercise interventions for healthy adults have shown promise for improving physical activity level [20,21,23], neuromuscular strength [24], physical performance [24], and reducing exercise-induced pain [25]. Exercise delivered via IVR may have the potential to improve adherence, enjoyment, and sustainability of exercise for clinical populations [26-29]. This may be in part due to heightened immersion leading to improved intrinsic motivation, enjoyment, and higher psychological engagement during exercise activity [23,30-32]. However, much of the previous review literature in this field has either combined immersive and nonimmersive technologies (eg, mobile apps) or primarily focused on behavioral and perceptual outcomes. Research findings regarding the clinical effectiveness of IVR exercise interventions remain scarce. A robust and broad investigation into the clinical effectiveness of these intervention types across clinical population groups is warranted. A detailed understanding of effectiveness is required to determine whether IVR could be promoted as an alternate or adjunct evidence-based exercise intervention by clinicians to improve health-related outcomes. Therefore, the objective of this systematic review was to assess the clinical effectiveness of IVR interventions using aerobic or anaerobic exercise approaches for health-related outcomes. Additionally, many feasibility considerations related to IVR exercise are not investigated systematically in previous literature. Feasibility information is vital to assess the potential for the implementation of the IVR exercise by clinicians. Therefore, this review also aimed to assess the feasibility of delivering these interventions to clinical cohorts through analysis of exercise session attendance and adherence, technological issues, motion sickness, safety, and participant experiences.

## Methods

### Overview

This systematic review incorporating meta-analysis is reported according to the expanded 2020 PRISMA (Preferred Reporting Items for Systematic Reviews and Meta-Analysis) checklist [33]. This review and protocol were registered under review number CRD420250650110 through PROSPERO International prospective register of systematic reviews on February 24, 2025.

### Terminology

IVR is a form of computer-human interface that allows the user to become immersed in a 3D, computer-generated environment while wearing an HMD [8].

Aerobic exercise is a structured and planned physical activity in which the body’s large muscles move in a rhythmic manner for a sustained period of time [34].

Anaerobic exercise is a structured and planned physical activity consisting of brief high-intensity bursts of exercise (such as strength training or fast boxing), where oxygen demand surpasses oxygen supply [34].

### Information Sources, Search Strategy, and Selection Process

The search strategy used in this systematic review is reported according to the PRISMA-S (PRISMA–Search) statement for reporting literature searches in systematic reviews [35]. Systematic searches were conducted by one reviewer (RCCB) across 4 databases from inception until January 14, 2025, and re-run on January 6, 2026. The databases searched included PubMed, Embase (Elsevier), Web of Science (Clarivate), and CINAHL Complete (EBSCOhost). Study registries were not searched for article identification purposes. Key search terms included “immersive virtual reality,” “immersive virtual reality exercise,” and “physical activity.” Search strategies for each database were cocreated by all members of the review team and are available in Multimedia Appendix 1. Search strategies from previous systematic reviews were not reused. Recursive and forward searching of reference lists for all included studies and other systematic reviews (those published in 2024 through Google Scholar) was completed. Covidence software (Veritas Health Innovation) was used to deduplicate records from the databases and complete screening procedures. Google Translate was used to translate reports from other languages to English. Only randomized controlled trials (RCTs) were included in this review. The non-RCT automation tool within Covidence software was applied to assist with the identification of ineligible study types prior to study screening. No additional information sources or search methods were used. Two reviewers (RCCB and MHR) contributed to study screening and selection. All disagreements in screening were resolved through consensus discussion.

### Eligibility Criteria

#### Participants

Included trials were not restricted by sex. Trials were included if participants had any of the following: (1) an acute health condition (eg, post–COVID-19 sequelae), (2) long-lasting chronic disease with persistent effects (eg, musculoskeletal or neurological conditions), (3) history of reconstructive or restorative surgery (eg, total knee arthroplasty), or (4) older adults. Studies including children or adolescents were not included.

#### Interventions

Participants must have undergone an IVR intervention incorporating aerobic or anaerobic exercise (supervised or unsupervised). Intervention duration must have been at least 3 weeks to allow for potential changes in effectiveness outcome measures. Trials were excluded if they used augmented reality (eg, smart glasses), non-IVR (eg, computer screen), semi-IVR (eg, driving simulator), an exergaming intervention not using an HMD (eg, Xbox Kinect), or were a nonexercise or balance-focused intervention.

#### Comparators

Exercising and nonexercising comparator groups were included. Single-arm trials were excluded.

#### Outcomes

Trials were included if they reported changes in continuous clinical effectiveness outcome measures. Authors were contacted for further information if eligible outcomes were measured but not reported. Eligible questionnaires presenting multiple domain scores were averaged for inclusion into meta-analyses (eg, quality of life questionnaires). Secondary feasibility outcomes were safety metrics (eg, adverse events), session attendance rates, exercise adherence, technological issues, motion sickness, and participant experiences.

#### Study Design

Completed RCTs were included. All other study designs (eg, nonrandomized trials, reviews, protocols, unpublished manuscripts, conference abstracts) were excluded. Studies were not restricted by language.

### Data Items and Collection Process

Data describing participant and trial characteristics, eligibility criteria, interventions, trial funding sources, clinical effectiveness outcomes, and feasibility outcomes were first extracted by 2 independent reviewers (RCCB and MHR) for 30% of trials (n=8) using Covidence and Microsoft Excel (Microsoft Corp) and tabulated into descriptive tables. After it was ascertained that there was 100% agreement between reviewers, one independent reviewer extracted data for the remainder of the trials (n=18; RCCB). Where further information was needed (n=3), authors were contacted via email by one reviewer (RCCB). Clinical effectiveness data were obtained for all reported timeframes in each individual trial. For studies presenting multiple assessments for the same clinical effectiveness domain, one was chosen for entry into the meta-analysis. All data in this review that were not reported in the original manuscripts were obtained via author correspondence.

### Risk of Bias and Certainty in Cumulative Estimates

The revised Cochrane Risk of Bias 2 tool (RoB2) [36] was used for quality appraisal for included trials. Study bias was assessed as “high,” “some concerns,” or “low” across 5 domains (randomization process, deviations from intended interventions, missing outcome data, measurement of the outcome, selection of the reported result) and facilitated by 2 independent reviewers (RCCB and MHR) for 28% of outcomes (n=15). Journal articles, protocol papers, and trial registrations were used in quality appraisal. Once consensus was achieved, the remaining outcomes were assessed by one reviewer (n=38; RCCB). An overall risk of bias for each outcome was determined using the RoB2 method [36]. Quality appraisal was based on published manuscripts. The certainty of evidence for clinical effectiveness outcomes was evaluated using the Grading of Recommendations, Assessment, Development, and Evaluation (GRADE) approach [37,38]. This was facilitated using GRADEPro GDT software [39], and assessed risk of bias, indirectness, inconsistency, imprecision, and other factors at the outcome level. The certainty of evidence was categorized as “high,” “moderate,” “low,” or “very low.” Outcome domains were downgraded if ≥50% of included trials presented issues in each criterion. Given the effect of exercise per se on clinical effectiveness outcomes, GRADE was facilitated according to the comparator group activity.

### Synthesis Methods and Meta-Analysis

Meta-analyses were completed using Comprehensive Meta-Analysis Software (version 4; Biostat) [40]. Clinical effectiveness domains reported in 3 or more trials with comparable control groups (ie, exercising or nonexercising) progressed to meta-analyses. Random effects meta-analyses with the Hartung-Knapp-Sidik-Jonkman adjustment allow for differences in treatment effect to be present and accounted for throughout the included trials with greater precision [41,42] and were conducted for the effects of the IVR exercise interventions for mobility and functional balance, condition severity (eg, WOMAC), quality of life, pain intensity, composite static and dynamic balance assessments, aerobic physical activity (device-measured), and static balance. Eligibility of outcome inclusion into individual meta-analyses was completed as part of the data extraction process. Double-counting of data for individual trials in meta-analyses did not occur. Where applicable, data were converted from 95% CIs into SDs for inclusion into the meta-analysis. For the meta-analyses, preintervention and postintervention means and SDs, and sample size per group were used. A within-group Cohen *d* effect size was calculated to estimate the change from baseline for each group. We used a plausible and conservative pre-post correlation of .5 measured within each comparison group [43,44]. For the effect size difference between groups, standardized mean differences (SMDs) were used. This was due to the differing forms of measurement for the above-listed clinical effectiveness domains. Prespecified levels of magnitude for SMD were set at .2 for small, .5 for moderate, and .8 for large [45]. The SMD and 95% CIs were calculated using random-effect meta-analyses with the inverse of variance. Prediction intervals were calculated for meta-analyses with 5 or more included studies. Statistical heterogeneity was assessed via the Q-test and prediction intervals, and between-study variability was calculated using the I^2^ statistic (0%‐25%=low, 26%‐74%=moderate, ≥75%=high) [46]. Prediction intervals were not calculated where statistical heterogeneity was low [47]. Data are presented in tables as per population group due to substantial heterogeneity identified. Small-study effects were assessed using the Egger test and visual inspection of funnel plots [48]. Additionally, the risk of bias due to missing results in meta-analyses was assessed through Egger test, funnel plots, and domains 3‐5 of the RoB2 tool. Analyses were grouped according to comparator group activity (exercising/nonexercising). Sensitivity analyses conducted included (1) removing individual trial results from the models to ascertain the effects on the overall results, and (2) running pre-post correlation at levels of 0.6, 0.7, 0.8, and 0.9 to assess influence on the overall result in line with Cochrane guidelines [43,44]. Subgroup analyses based on population were not completed due to the scarcity of common data.

### Ethical Considerations

This systematic review uses data that are publicly available through previous literature and did not require research ethical approval. The review was registered through PROSPERO International prospective register of systematic reviews on February 24, 2025 (CRD420250650110).

## Results

### Overview

**Figure 1.**
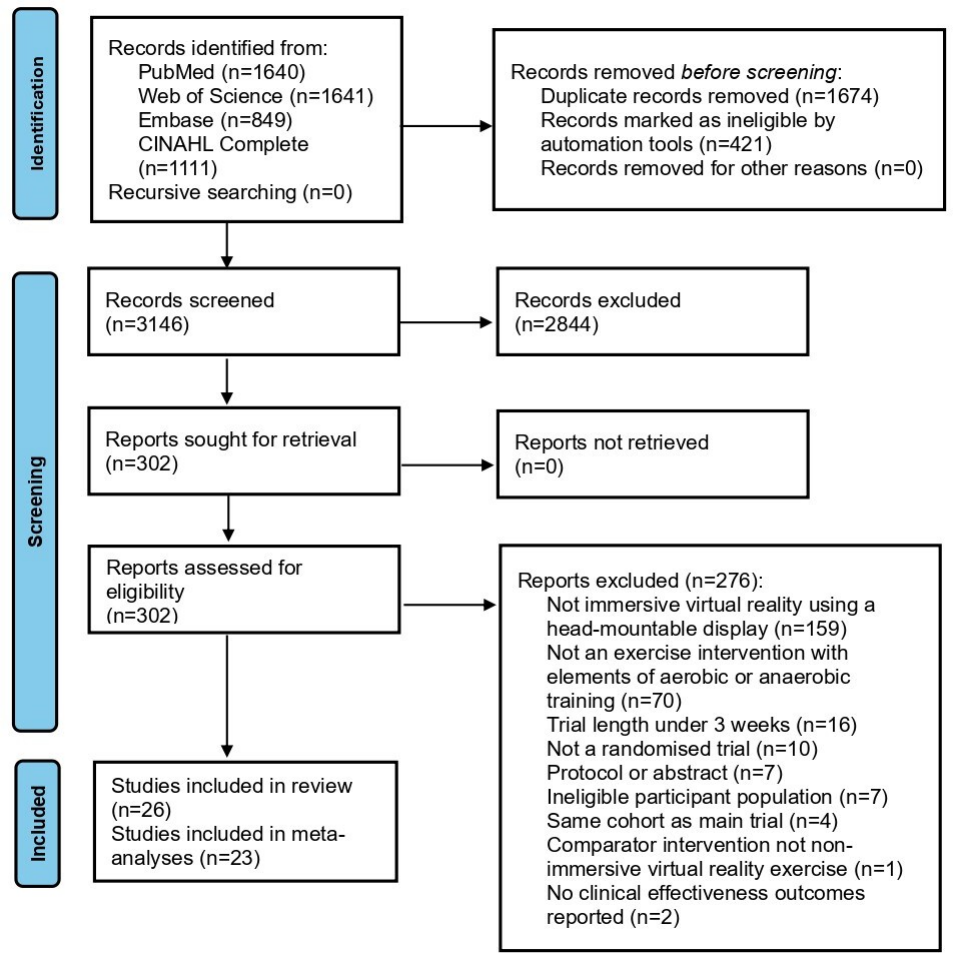
PRISMA (Preferred Reporting Items for Systematic reviews and Meta-Analyses) flow diagram.

Figure 1 displays that 5241 records were identified in the initial search. A total of 26 trials were included, with 23 progressing to meta-analyses.

### Trial Characteristics

Trial characteristics are displayed in Table 1. Trials were conducted from 2021 to 2025 in Spain [28,49-51] (n=4), China [29,52,53] (n=3), Germany [54-56] (n=3), India [57-59] (n=3), and other countries [60-72] (n=13). A total of 846 people participated across all trials. Average sample size across trials was 33.5 (SD 18.0) participants, and mean age was 60.7 (SD 15.8) years. Trials included participants with neurological disorders [49-51,59,70,71], older adults [28,52,56,60,61,63,64,67-69], musculoskeletal conditions [29,55,57,58,62,66], cardiopulmonary diseases [53,65], cancer [54], and metabolic conditions [72]. The most common population group was older adults, accounting for 349 (41%) of all participants.

**Table 1.** Trial characteristics, including design, population, setting, provider, follow-up points, and funding source.

Reference, year, country, population	Trial design	Participants (women/men)		Age (years), mean (SD)		Setting	Provider	Follow up points	Trial funding source
		Intervention	Control	Intervention	Control				
Older adults									
Barsasella et al, 2021, Taiwan, Older adults [60]	RCT^a^	29 (25/4)	31 (21/10)	71.7 (7.4)	70.7 (7.4)	Community	Researcher	6 weeks	Ministry of Science and Technology, Taiwan; Taipei Medical University; Wanfang hospital; Ministry of Education
Campo-Prieto et al, 2022, Spain, Older adults [28]	Feasibility RCT	13 (11/2)	11 (10/1)	85.08 (8.48)	84.82 (8.10)	Community	NR^b^	10 weeks	Galician Government (Xunta de Galicia) Predoctoral fellowship
Drazich et al, 2023, United States of America, Older adults [61]	Pilot RCT	10 (9/1)	9 (6/3)	76.4 (7.6)	71.4 (3.9)	Community	Researcher	8 weeks	Sigma Theta Tau international honor society of nurses; Southern Nursing Research Society
Kershner et al, 2024, United States of America, Older adults [63]	Pilot RCT	5 (3/2)	4 (3/1)	66.00 (63.60‐68.40)^c^^d^	65.50 (63.25‐67.75)^d^	Community	Behavioral coach	4 weeks	Wells Fargo Faculty Scholar Award
Kwan et al, 2021, China, Older adults [52]	RCT	9 (8/1)	8 (7/1)	73 (7.5)^c^	77.5 (15.3)	Community	Researcher	8 weeks	Innovation and Technology Fund for Better Living from The Hong Kong Polytechnic University
Lima Rêbelo et al, 2021, Brazil, Older adults [64]	RCT	20 (16/4)	17 (15/2)	69.25 (5.67)	71.41 (5.94)	Community	Researcher	8 weeks	No funding source
Parmak et al, 2025, Northern Cyprus, Older adults [69]	RCT	22 (18/4)	22 (18/4)	71.14 (4.83)	75.36 (9.16)	Community	Physiotherapist	8 weeks	No funding source
Vorwerg-Gall et al, 2024, Germany, Older adults [56]	Pilot RCT	23 (14/9)	12 (10/2)	69 (4.7)	66 (5.2)	Community	Physiotherapist	6 weeks	Federal Ministry of Education and Research Germany
Yalfani et al, 2024, Iran, Older adults [67]	RCT	12 (12/0)	12 (12/0)	68.25 (2.92)	67.08 (2.9)	Laboratory	NR	8 weeks	No funding source
Zak et al, 2024, Poland, Older women [68]	RCT	40 (40/0)	40 (40/0)	76.7 (2.21)	76.75 (1.77)	NR	Physiotherapist	6 and 9 weeks	Chancellor’s Grant: Jan Kochanowski University of Kielce, Poland
Neurological									
An and Park, 2022, South Korea, Incomplete spinal cord injury [71]	RCT	20 (8/12)	20 (9/11)	42.27 (5.51)	43.00 (7.15)	Community	Physiotherapist	4 weeks	NR
Peláez-Vélez et al, 2023, Spain, Stroke [49]	Pilot RCT	12 (3/9)	12 (5/7)	51.91 (18.58)	59.58 (15.97)	Hospital	Physiotherapist	6 weeks	No funding source
Ramos et al, 2025, Australia, Developmental disability [70]	RCT	13 (3/10)	8 (3/5)	36.0 (12.0)	36.0 (13.0)	Community	Exercise Physiologist	8 weeks	University of South Australia
Rodriguez-Fuentes et al, 2024, Spain (a), Parkinson disease [50]	RCT	30 (11/19)	22 (11/11)	70.87 (6.67)	70.59 (6.67)	NR	NR	12 weeks	Intramural Call for Biomedical Research Projects 2022, from the Galicia Sur Health Research Institute
Rodriguez-Fuentes et al, 2024, Spain (b), Multiple sclerosis [51]	Feasibility RCT	8 (6/2)	10 (7/3)	41.13 (4.88)	48.2 (5.40)	NR	Physiotherapist	8 weeks	Colexio Oficial de Fisioterapeutas de Galicia (Official Physical Therapy Council of Galician, Spain), section Research Grant 2023
Vishnuram et al, 2024, India, Stroke [59]	Pilot RCT	4 (NR)	4 (NR)	NR	NR	NR	Physiotherapist	4 weeks	No funding source
Musculoskeletal									
Gsangaga et al, 2023, Malaysia, Postanterior cruciate ligament reconstruction [62]	RCT	15 (5/10)	15 (2/13)	28.6 (NR)	25.1 (NR)	Hospital	Physiotherapist	26 weeks	Dana Fundamental PPUKM
Lo et al, 2024, China, Knee osteoarthritis [29]	Pilot RCT	15 (10/5)	15 (13/2)	63 (60-67)^c^^d^	64 (62-65)^d^	Community	Physiotherapist	12 weeks	Hong Kong Jockey Club Charities Trust
Naqvi et al, 2022, India, Distal radius fracture [57]	Pilot RCT	10 (NR)	10 (NR)	NR	NR	Hospital	NR	2 and 4 weeks	No funding source
Nishitha et al, 2024, India, Total knee arthroplasty [58]	RCT	18 (NR)	18 (NR)	NR	NR	NR	Physiotherapist	2, 3, 4, 9, and 12 weeks	NR
Stamm et al, 2022, Germany, Chronic low back pain [55]	Pilot RCT	11 (8/3)	11 (6/5)	75 (5.8)	75.5 (4.39)	Laboratory	Physiotherapist	4 weeks	German Federal Ministry of Education and Research
Tuck et al (2022), New Zealand, Chronic musculoskeletal pain [66]^d^	Mixed methods RCT	10 (8/2)	10 (5/5)	41.3 (17.7)	38.7 (15.3)	Hospital	Physiotherapist	6 weeks	Auckland University of Technology
Cardiopulmonary									
Rutkowski et al, 2022, Poland, Post COVID-19 [65]	RCT	16 (NR)	16 (NR)	NR	NR	Hospital	Physiotherapist	3 weeks	The Polish National Agency for Academic Exchange (Urgency Grant)
Wang et al, 2023, China, Coronary heart disease [53]	RCT	18 (9/9)	18 (8/10)	72.50 (6.16)	72.61 (5.45)	Hospital	Physiotherapist	12 weeks	NR
Cancer									
Schrempf et al, 2023, Germany, Colorectal cancer [54]	Pilot RCT	31 (12/19)	31 (13/18)	60.4 (9.5)	60.9 (9.7)	Hospital	Hospital staff or doctoral students	1 and 4 weeks	University of Augsburg
Metabolic									
Seo et al, 2023, South Korea, Overweight middle-aged women^e^ [72]	RCT	23 (23/0)	23 (23/0)	47.74 (5.50)	48.26 (7.56)	Hospital	Researcher	4 and 8 weeks	National Research Foundation of Korea grant (Korean Government)

a RCT: randomized controlled trial.

b NR: not reported.

c The third group consists of the same participants as the control and is not included in this review.

d Median (IQR).

e TG of participants is 24 (24/0) and age is 49.00 (6.77).

### Intervention Characteristics

Details of the virtual reality interventions are summarized in Table 2. Aerobic interventions were used in 9 trials [50,52,54,57,60,61,63,65,72], anaerobic interventions in 4 [29,59,68,71], and a mix of aerobic/anaerobic in 13 [28,49,51,53,55,56,58,62,64,66,67,69,70]. Exercise intensity metrics were reported in 9 trials [50,53-56,63,65,70,72]. Dedicated IVR systems were used in 19 trials [28,49-57,60-67,69], 4 used smartphones in combination with a head mountable stand [29,59,68,72], and 3 did not specify [58,70,71]. Physiotherapists were the most common intervention provider (14/26, 54%). Intervention duration ranged from 30 days to 26 weeks, with 6‐8 weeks being the most common (14/26, 54%). Most participants in virtual reality groups were supervised in person (19/26, 73%).

**Table 2.** Intervention characteristics, including type, immersive virtual reality system used, game type, additional equipment, sessions per week, intensity, session length, length of intervention, location, and cointerventions.

Reference	Intervention	IVR system	Game type	Additional equipment	Sessions/wk	Intensity	Session length (mins)	Length (wks)	Location	Cointerventions
Older adults										
Barsasella et al, 2021 [60]	I^a^: Aerobic C^b^: NA^c^	HTC Vive	Commercial	None	I: 2 C: NA	I: NR^d^ C: NA	I: 15 C: NA	I & C: 6	I: In-person (S^e^) C: NA	I & C: NA
Campo-Prieto et al, 2022 [28]	I: Aerobic & Anaerobic (boxing) C: NA	HTC Vive Pro	Commercial	None	I: 3 C: NA	I: NR C: NA	I: 6 C: NA	I & C: 10	I & C: In-person (S)	I: IVR^g^ given in addition to UC^h^ C: NA
Drazich et al, 2023 [61]	I: Aerobic C: NA	Oculus Quest 2	Commercial	Nordic Track R35 Recumbent Cycle Ergometer	I: 2 C: NA	I: Patient-led (no metric) C: NA	I: 40 C: NA	I & C: 8	I: In-person (S) C: NA	I: Received control intervention in addition C: Education program
Kershner et al, 2024 [63]	I & C: Aerobic	Meta Quest 2	Commercial	None	I & C: Patient-led	I: MVPA^i^ (>70% max HR^j^) C: MVPA (>70% max HR)	I & C: Pt-led	I & C: 4	I: Home (S) C: Home (US^f^)	I & C: 8 x individual and 4 x group meetings with behavior coach to discuss MVPA achievement
Kwan et al, 2021 [52]	I & C: Aerobic	HTC Vive Focus Plus	Bespoke	Under-desk ergometer	I & C: 2	I & C: Patient-led (no metric)	I & C: 15	I & C: 8	I & C: In-person (S)	I & C: Additional cognitive training
Lima Rêbelo et al, 2021 [64]	I: Aerobic & Anaerobic (boxing & strength) C: Balance	Oculus Rift	Commercial	None	I & C: 2	I: NR C: Progressive (no metric)	I & C: 50	I & C: 8	I & C: In-person (S)	I & C: NA
Parmak et al, 2025 [69]	I: Aerobic & Anaerobic (boxing) C: Balance, Anaerobic (strength)	Oculus Meta Quest 2	Commercial	None	I & C: 3	I: Patient-led (no metric)	I & C: 35	I & C: 8	I: In-Person (S) C: Home (US)	I & C: NA
Vorwerg-Gall et al, 2024 [56]	I & C: Aerobic & Anaerobic (strength)	HTC Vive Pro	Bespoke	Dumbbells with hand location tracking	I & C: 2	I & C: Aerobic: 40%‐60% HRR^k^ Anaerobic (strength): 2‐3 sets for 20‐30 reps^l^	I & C: 30	I & C: 6	I & C: In-person (S)	I & C: Breathing exercises
Yalfani et al, 2024 [67]	I: Aerobic & Anaerobic (boxing) C: NA	HTC Vive	Commercial	None	I: 3 C: NA	I: NR C: NA	I: 30 C: NA	I & C: 8	I: In-person (S) C: NA	I: NA C: No rehabilitation intervention received
Zak et al, 2024 [68]	I & C: Anaerobic (strength)	VR ONE plus-ZEISS (smartphone)	Commercial	None	I & C: 3	I & C: NR	I & C: 60	I & C: 6	I & C: In-person (S)	I: Given control intervention in addition C: NA
Neurological										
An and Park, 2022 [71]	I & C: Anaerobic (strength)	NR	Bespoke	Lower limb sensors	I & C: 3	I & C: NR	I & C: 30	I & C: 4	I & C: NR	I & C: NA
Peláez-Vélez et al, 2023 [49]	I & C: Aerobic & Anaerobic (strength)	Oculus Quest 2	Bespoke	None	I: 5 (+3 IVR) C: 5	I & C: NR	I: 60 (+30 for IVR) C: 60	I & C: 6	I & C: In-person (S)	I: Received control intervention C: NA
Ramos et al, 2025 [70]	I: Aerobic & Anaerobic (boxing) C: NA	NR	Commercial	None	I: 3 C: NA	I: *Aerobic & Anaerobic (boxing*): Borg RPE scale (NR) C: NA	I: 60 C: NA	I & C: 8	I: In-person (S) C: NA	I & C: NA
Rodriguez-Fuentes et al, 2024 (a) [50]	I & C: Aerobic	Meta Quest 2	Commercial	Cycle ergometer with IVR connectivity	I & C: 2	I & C: 80‐90 RPM^m^; 70%‐80% of Max HR, Borg RPE^n^ 8-9/10	I & C: 25	I & C: 12	I & C: In-person (S)	I & C: NA
Rodriguez-Fuentes et al, 2024 (b) [51]	I: Aerobic & Anaerobic (boxing) C: NR (“Exercising”)	Oculus Quest 3	Commercial	None	I: 2 (+ C) C: NR	I & C: NR	I: 6 C: NR	I & C: 8	I & C: In-person (S)	I: Received control intervention in addition to VR C: Usual activities conducted in-center (NR)
Vishnuram et al, 2024 [59]	I & C: Anaerobic (strength)	JioDive 360^0^ (smartphone)	NR	None	I: 14 (+ NR IVR frequency) C: 14	I & C: NR	I: 30 (+30‐90 for IVR) C: 30	I & C: 4	I & C: NR	I: Received C intervention in addition + PNM^o^
Musculoskeletal										
Gsangaga et al, 2023 [62]	I & C: Aerobic & Anaerobic (boxing)	PlayStation^®^ VR	Commercial	None	I: 14 (+ IVR every 2) C: 14	I & C: NR	I: NR (+30 IVR) C: NR	I: 26 (first 12 C) C: 26	I & C: In-person (S) & Home (US)	I: Received C in addition to IVR C: NA
Lo et al, 2024 [29]	I & C: Anaerobic (strength)	VR Shinecon 5.0 (smartphone)	Bespoke	Lower limb sensors with IVR	I & C: 5	I & C: NR	I & C: 30	I & C: 12	I & C: Home (US)	I & C: NA
Naqvi et al, 2022 [57]	I: Aerobic C: NR	Oculus Quest	Commercial	NR	I & C: 5	I & C: NR	I & C: 60	I & C: 4	I & C: NR (S)	I & C: NA
Nishitha et al, 2024 [58]	I: Aerobic & Anaerobic (strength) C: Anaerobic (strength)	NR	NR	None	I & C: 4	I: NR C: “High” (metric NR)	I: 40 C: NR	I & C: 12	I & C: In-person (S)	I: Nonimmersive VR exercise program included C: NA
Stamm et al, 2022 [55]	I: Aerobic & Anaerobic (strength) C: Anaerobic (strength)	HTC Vive	Bespoke	None	I & C: 3	I: 75% HRR C: NR	I & C: 30	I & C: 4	I & C: In-person (S)	I & C: Education sessions
Tuck et al, 2022 [66]	I & C: Aerobic, anaerobic (strength & boxing) TG: NA	HTC Vive	Commercial	None	I: 2 C: NR TG^p^: NA	I & C: NR TG: NA	I & C: NR TG: NA	I, C & TG: 6	I: In-person (S) C: Home (US) TG: NA	I & C: Education sessions TG: Waitlist control
Cardiopulmonary										
Rutkowski et al, 2022 [65]	I & C: Aerobic	VR TierOne	Bespoke	COSMED cycle ergometer with IVR connectivity	I & C: 7	I & C: 20%‐80% APHRM^q^	I & C: NR	I & C: 3	I & C: In-person (S)	I: Additional relaxation exercises in IVR C: NA
Wang et al, 2023 [53]	I: Aerobic & Anaerobic (boxing) C: NA	Pico Neo 3	Commercial	None	I: 7 C: NA	I: “low-medium” intensity (Borg RPE 12/20; 60%‐70% APHRM) C: NA	I: 20‐30 C: NA	I & C: 12	I: In-person (S) C: NA	I: Breathing exercises C: Education and follow-ups
Cancer										
Schrempf et al, 2023 [54]	I: Aerobic C: NA	Oculus Quest 2	Commercial	None	I & C: 7	I: “Moderate” 50%‐70% APHRM C: NA	I & C: 30	I & C: 30 days	I: In-person (S & US) C: In-person (S)	I: NA C: Nonexercise physiotherapy treatment
Metabolic										
Seo et al, 2023 [72]	I & TG: Aerobic C: NA	Smartphone HMD^r^	Commercial	Cycle ergometer with sensors for IVR	I & TG: 3‐5 C: NA	I & TG: “Low” intensity (talk test) C: NA	I & TG: 50 C: NA	I, C & TG: 8	I & TG: Home (US) C: NA	I & TG: NA C: Daily activities with no intervention

a I: intervention.

b C: control.

c NA: not applicable.

d NR: not reported.

e S: supervised.

f US: unsupervised.

g IVR: immersive virtual reality.

h UC: usual care.

i MVPA: moderate-vigorous physical activity.

j HR: heart rate.

k HRR: heart rate reserve.

l Reps: repetitions.

m RPM: revolutions per minute.

n RPE: rating of perceived exertion.

o PNM: peripheral nerve mobilization.

p TG: third group.

q APHRM: age-predicated heart rate maximum.

r HMD: head-mounted display.

### Outcomes

Data on all 30 clinical effectiveness outcome domains identified in this review are presented in Table S1 in Multimedia Appendix 1. Clinical effectiveness outcome domains progressing to meta-analyses included mobility and functional balance, condition severity, quality of life, functional leg strength, pain intensity, composite static and dynamic balance assessments, aerobic physical activity (device-measured), and static balance. Outcome domains included in meta-analyses are summarized in Table 3.

**Table 3.** Outcomes included in meta-analyses from included trials.

Reference	Intervention, mean (SD)		Control, mean (SD)	
	Baseline	Postintervention	Baseline	Postintervention
Older adults				
Barsasella et al, 2021 [60]				
Functional leg strength: 30 s Sit to Stand Test (repetitions)	21.6 (9.0)	22.0 (7.8)	19.8 (7.3)	19.8 (7.2)
Mobility and functional balance: 8-Foot Up and Go Tests	7.5 (2.7)	6.5 (2.5)^d^	7.0 (2.7)	5.7 (1.7)^d^
Static balance: Single Leg Stances	16.6 (9.6)	15.8 (10.1)	14.5 (10.4)	15.1 (9.7)
Campo-Prieto et al, 2022 [28]				
Functional leg strength: 5 × Sit to Stand Tests	15.6 (4.5)	13.8 (3.5)^c^	21.2 (12.6)	25.6 (14.2)^d^
Mobility and functional balance: Timed Up and Go Tests	17.9 (6.4)	19.0 (6.6)	23.2 (9.3)	26.3 (11.8)^d^
Quality of life: Short Form-12 Health Survey (SF12): Mental Component Summary (score)	48.8 (8.7)	53.4 (8.7)	50.4 (10.5)	55.2 (9.5)
Quality of life: Short Form-12 Health Survey (SF12): Physical Component Summary (score)	48.8 (9.5)	48.5 (9.0)^c^	42.1 (12.7)	44.4 (9.9)
Kershner et al, 2024 [63]				
Aerobic physical activity (device-measured): Weekly Total Physical Activity (min)	773.8 (243.0)	807.77 (189.6)	322.0 (137.5)	705.5 (244.1)
Lima Rêbelo et al, 2021 [64]				
Mobility and functional balance: Timed Up and Go Test (s)^b^	NR	−1.71 (2.3)^c^	NR^e^	−1.22 (3.6)
Static balance: Clinical Test of Sensory Interaction and Balance C.1 (score)^b^	NR	1.0 (6.8)	NR	1.5 (6.9)
Static balance: Clinical Test of Sensory Interaction and Balance C.2 (score)^b^	NR	2.6 (8.3)	NR	4.1 (10.6)^c^
Static balance: Clinical Test of Sensory Interaction and Balance C.3 (score)^b^	NR	3.9 (11.6)	NR	7.3 (12.1)
Static balance: Clinical Test of Sensory Interaction and Balance C.4 (score)^b^	NR	8.0 (11.8)^d^	NR	13.2 (14.6)^d^
Parmak et al, 2025 [69]				
Composite static and dynamic balance: Fullerton Advanced Balance Scale (score)	28.8 (4.7)	34.5 (3.8)^c^	27.3 (6.6)	30.2 (6.1)
Functional leg strength: 30 s Sit to Stand test (repetitions)	12.1 (3.2)	13.9 (2.4)^c^	10.7 (2.9)	11.8 (2.7)
Mobility and functional balance: Eight-Step Walk Tests	5.9 (0.9)	5.6 (0.9)	6.1 (1.1)	5.7 (1.1)
Quality of Life: World Health Organization Quality of Life Instrument: Older Adults (score)	80.3 (8.2)	83.9 (9.8)	74.4 (10.0)	77.6 (11.0)
Vorwerg-Gall et al, 2024 [56]				
Functional leg strength: 5 × Sit to Stand Tests^b^	NR	−1.0 (1.4)^d^	NR	−1.5 (1.9)^d^
Yalfani et al, 2024 [67]				
Functional leg strength: 30 s Sit to Stand Test (repetitions)	10.3 (1.8)	12.1 (1.6)^c^^,d^	10.1 (1.5)	9.3 (2.1)
Static balance: Center of Pressure Anteroposterior Sway (N/cm^2^)^f^	18.5 (7.5)	11.3 (4.3)^c^^,d^	12.3 (2.64)	14.3 (3.7)
Static balance: Center of Pressure Mediolateral Sway (N/cm^2^)	8.9 (2.3)	6.6 (0.5)^c^^,d^	7.7 (2.4)	8.3 (2.4)
Mobility and functional balance: Timed Up and Go Tests	12.6 (2.2)	9.6 (1.6)^c^	11.7 (2.1)	11.3 (2.2)
Zak et al, 2024 [68]				
Mobility and functional balance: Timed Up and Go Tests	14.0 (1.7)	12.6 (1.3)^d^	13.5 (1.1)	11.4 (1.1)^c^^,d^
Composite static and dynamic balance: Berg Balance Scale (score)	39.7 (1.3)	41.9 (2.4)^d^	39.3 (0.9)	42.6 (2.8)^c^^,d^
Neurological				
An and Park, 2022 [71]				
Functional leg strength: 5 × Sit to Stand Tests	54.1 (6.7)	35.2 (7.8)^c^^,d^	52.6 (6.5)	37.5 (10.0)^c^
Mobility and functional balance: Timed Up and Go Tests	23.2 (4.1)	14.5 (4.4)^c^^,d^	22.3 (3.2)	15.3 (4.7)^d^
Peláez-Vélez et al, 2023 [49]				
Composite static and dynamic balance: Tinetti Test Gait (score)	5.4 (3.0)	9.2 (2.9)^d^	3.6 (4.5)	5.8 (4.0)
Composite static and dynamic balance: Tinetti Test Balance (score)	8.6 (4.1)	13.6 (3.1)^d^	6.5 (6.1)	9.1 (5.7)
Composite static and dynamic balance: Berg Balance Scale (score)	27.0 (15.9)	46.0 (13.1)^d^	21.3 (22.8)	28.9 (20.4)
Condition severity: Motricity Index (score)	67.1 (31.7)	84.0 (23.1)^d^	72.7 (37.7)	75.7 (36.7)
Ramos et al, 2025 [70]				
Mobility and functional balance: Timed Up and Go Tests	8.5 (1.9)	7.9 (1.6)	8.7 (1.9)	8.9 (2.3)
Functional leg strength: 30 s Sit to Stand Test (repetitions)	11.0 (3.0)	11.0 (3.0)	9.0 (2.0)	11.0 (3.0)
Rodriguez-Fuentes et al, 2024 (a) [50]				
Functional leg strength: 5 × Sit to Stand Tests	14.3 (5.4)	13.8 (6.7)	13.1 (3.0)	13.2 (4.5)
Mobility and functional balance: Timed Up and Go Tests	14.1 (18.3)	10.2 (6.4)^b^	12.5 (9.2)	13.7 (4.5)
Composite static and dynamic balance: Tinetti Test (score)	22.4 (4.9)	26.4 (3.5)^c^^,d^	25.0 (3.9)	25.5 (3.6)
Quality of life: Parkinson disease Questionnaire (score)	7.0 (5.6)	6.4 (4.9)^c^	10.8 (7.5)	9.7 (6.5)
Condition severity: Movement Disorders Society Modified Unified Parkinson Disease Rating Scale (score)	21.9 (18.8)	15.1 (9.8)^d^	42.7 (31.3)	34.8 (23.4)
Rodriguez-Fuentes et al, 2024 (b) [51]				
Composite static and dynamic balance: Tinetti Test (score)	26.1 (2.1)	26.6 (1.5)	25.2 (2.9)	25.4 (2.3)
Functional leg strength: 5 × Sit to Stand Tests	14.4 (4.5)	12.8 (3.8)^d^	12.7 (4.7)	12.1 (2.8)
Mobility and functional balance: Timed Up and Go Tests	10.7 (5.6)	9.2 (4.7)^d^	8.0 (1.9)	8.7 (2.3)
Musculoskeletal				
Gsangaya et al, 2023 [62]				
Pain intensity: Numerical Pain Rating Scale (score)	4.6 (0.8)	0.4 (0.5)^c^^,d^	4.8 (0.9)	0.9 (0.7)^d^
Condition severity: International Knee Documentation Committee Score (score)	82.9 (3.2)	93.9 (1.0)^b^^,d^	85.0 (3.8)	92.9 (1.2)^d^
Lo et al, 2024 [29]				
Pain intensity: Numerical Pain Rating Scale (score)	5.9 (1.9)	4.8 (1.8)	4.8 (1.5)	4.6 (2.4)
Condition severity: Western Ontario and McMaster Universities Osteoarthritis Index (score)	902.9 (454.8)	791.3 (425.1)	752.0 (549.3)	742.4 (510.5)
Quality of life: EuroQol Visual Analog Scale (score)	62.0 (19.6)	71.6 (12.8)	67.3 (17.2)	67.7 (17.4)
Aerobic physical activity (device-measured): MET/d (score)	34.2 (1.2)	34.4 (1.3)	35.0 (1.4)	34.9 (2.0)
Naqvi et al, 2022 [57]				
Pain intensity: Visual Analog Scale (score)	7.0 (0.7)	1.77 (0.4)^c^^,d^	7.5 (0.5)	4.2 (0.3)^d^
Condition severity: Disabilities of the Arm, Shoulder, and Hand Questionnaire (score)	80.2 (1.7)	13.0 (1.7)^c^^,d^	80.7 (2.8)	28.1 (6.6)^d^
Nishitha et al, 2024 [58]				
Pain intensity: Numerical Pain Rating Scale (score)	3.4 (0.5)	1.5 (0.5)^b^	3.8 (0.4)	2.3 (0.5)
Condition severity: Western Ontario and McMaster Universities Osteoarthritis Index (score)	51.2 (1.3)	14.9 (1.0)^b^	62.1 (1.6)	19.0 (2.0)
Mobility and functional balance: Timed Up and Go Tests	23.2 (1.6)	8.6 (1.1)^b^	28.7 (1.3)	12.1 (0.9)
Stamm et al, 2022 [55]				
Pain intensity: Numerical Pain Rating Scale (score)	3.6 (2.4)	2.9 (2.0)	2.9 (2.4)	1.6 (1.5)
Condition severity: Hannover Functional Ability Questionnaire (score)	73.1 (10.6)	81.8 (11.2)^d^	69.8 (16.8)	72.7 (15.7)
Quality of life: Short Form-12 Health Survey (SF12): Mental Component Summary (score)	46.4 (10.6)	48.4 (7.1)	50.3 (7.7)	56.2 (4.8)^d^
Quality of life: Short Form-12 Health Survey (SF12): Physical Component Summary (score)	41.0 (7.8)	39.3 (8.0)	35.9 (7.9)	37.8 (7.3)
Tuck et al, 2022 [66]				
Pain intensity: Brief Pain Inventory (score)^a^^b^	8.4 (1.8)	−1.0 (0.9)	8.1 (1.2)	−0.2 (2.3)
Aerobic physical activity (device-measured): Daily Active Minutes (mins)^a^^b^	NA	19.5 (64.5)	NA	−21.1 (91.5)
Cardiopulmonary				
Rutkowski et al, 2022 [65]				
Quality of life: World Health Organization Quality of Life Scale (score)	61.6 (16.2)	62.8 (14.1)	59.7 (17.9)	62.9 (16.8)^d^
Wang et al, 2023 [53]				
Quality of life: 36-Item Short Form Survey (score)	66.6 (12.0)	79.7 (11.6)^d^	66.3 (11.5)	76.2 (11.9)
Cancer				
Schrempf et al, 2023 [54]				
Quality of life: EQ-5D-5L (score)	0.9 (0.1)	0.8 (0.2)	0.9 (0.1)	0.8 (0.2)

a Exercising control group.

b Change from baseline.

c Between-group difference.

d Within-group difference.

e NR: Not reported.

f N/cm2: Newtons per square centimeter.

### Meta Analyses

**Figure 2.**
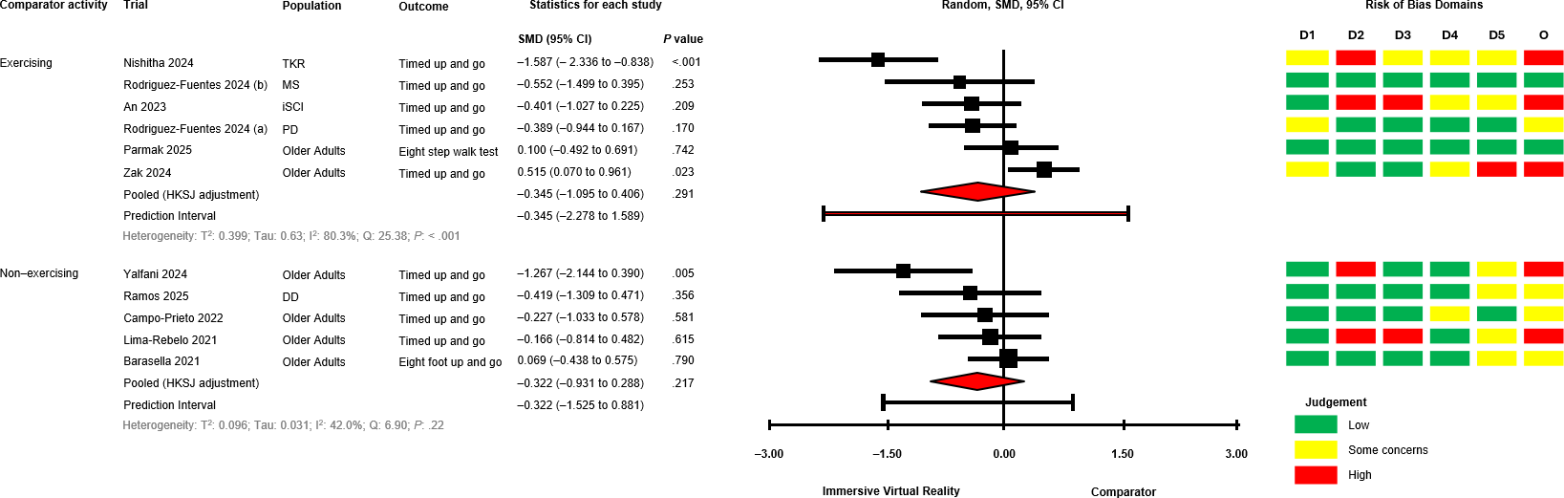
Between-group random effects meta-analysis of mobility and functional balance assessments, grouped by comparator group activity [28,50,51,58,60,64,67-71]. D1: Domain 1; D2: Domain 2; D3: Domain 3; D4: Domain 4; D5: Domain 5; DD: Developmental Disability; HKSJ: Hartung-Knapp-Sidik-Jonkman; iSCI: Incomplete Spinal Cord Injury; MS: Multiple Sclerosis; O: Overall; PD: Parkinson disease; SMD: standardized mean difference; TKR: Total Knee Replacement.

**Figure 3.**
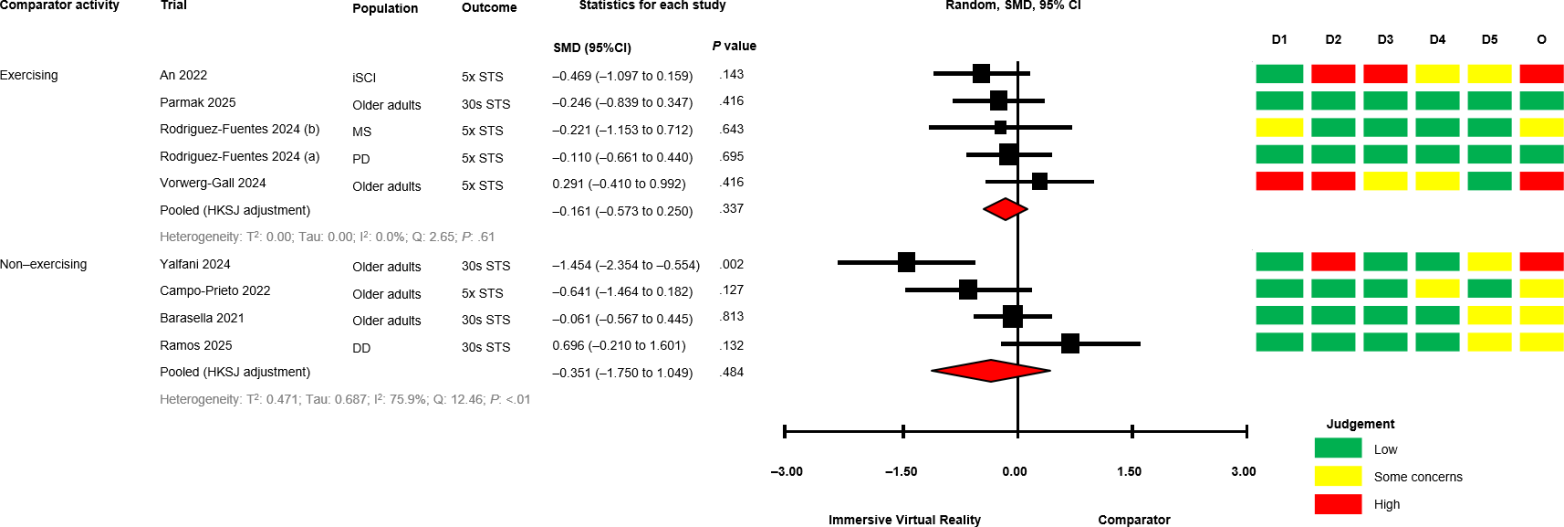
Between-group random effects meta-analysis of functional leg strength assessments, grouped by comparator group activity [28,50,51,56,60,67,69-71]. 30 s STS: 30-seconds Sit to Stand Test; 5 × STS: 5-times Sit to Stand Test; D1: Domain 1; D2: Domain 2; D3: Domain 3; D4: Domain 4; D5: Domain 5; DD: Developmental Disability; HKSJ: Hartung-Knapp-Sidik-Jonkman; iSCI: Incomplete Spinal Cord Injury; MS: Multiple Sclerosis; O: Overall; PD: Parkinson disease; SMD: standardized mean difference.

**Figure 4.**
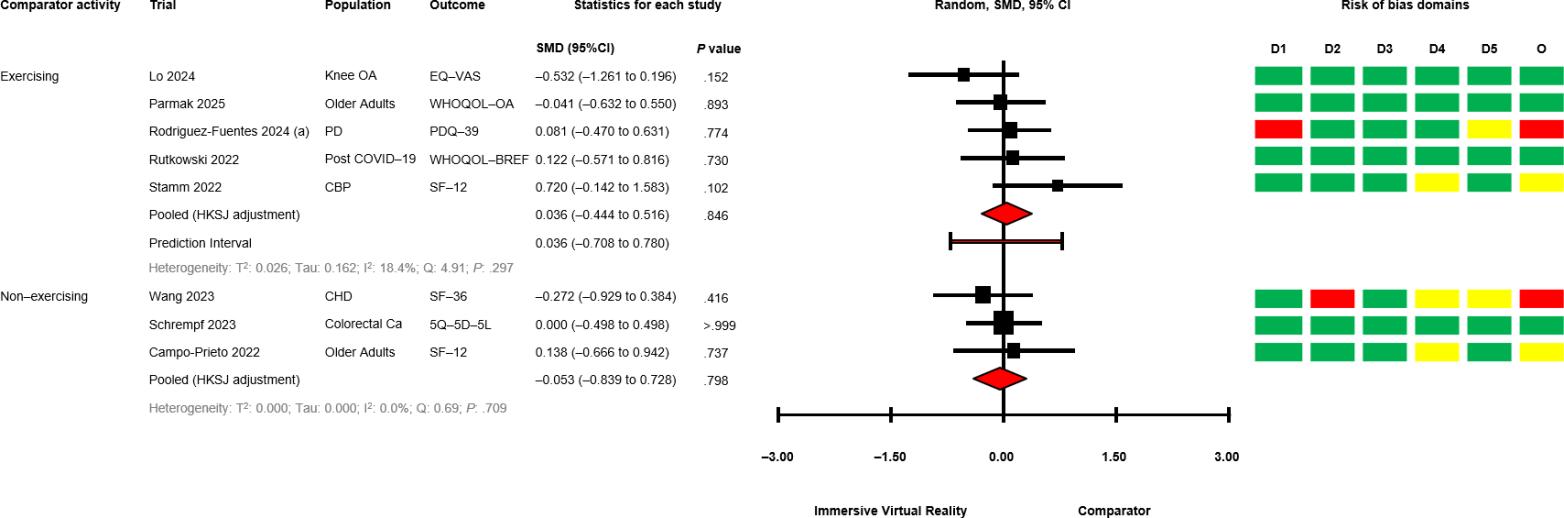
Between-group random effects meta-analysis of quality of life assessments, grouped by comparator group activity [28,29,50,53-55,65,69]. Ca: Cancer; CBP: Chronic Back Pain; CHD: Coronary Heart Disease; D1: Domain 1; D2: Domain 2; D3: Domain 3; D4: Domain 4; D5: Domain 5; EQ-VAS: EQ–Visual Analog Scale; HKSJ: Hartung-Knapp-Sidik-Jonkman; O: Overall; OA: Osteoarthritis; PD: Parkinson disease; PDQ-39: Parkinson disease questionnaire; SF-12: Short Form-12 Health Survey; SF-36: 36-Item Short Form Survey; SMD: standardized mean difference; WHOQOL-BREF: World Health Organization quality of life scale; WHOQOL-OA: World Health Organization quality of life instrument: Older adults.

**Figure 5.**
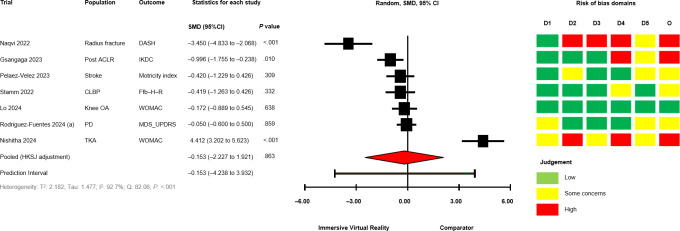
Between-group random effects meta-analysis of condition severity assessments for trials with an exercising comparator group [29,49,50,55,57,58,62]. ACLR: Anterior Cruciate Ligament Reconstruction; CLBP: Chronic Lower Back Pain; D1: Domain 1; D2: Domain 2; D3: Domain 3; D4: Domain 4; D5: Domain 5; DASH: Disability of the Arm, Shoulder, and Hand Questionnaire; Ffb-H-R: Hannover Functional Ability Questionnaire; HKSJ: Hartung-Knapp-Sidik-Jonkman; IKDC: International Knee Documentation Committee Score; MDS_UPDRS: Movement Disorders Society Modified Unified Parkinson Disease Rating Scale; O: Overall; OA: Osteoarthritis; PD: Parkinson disease; SMD: standardized mean difference; TKA: Total Knee Arthroplasty; WOMAC: Western Ontario and McMaster Universities Osteoarthritis Index.

**Figure 6.**
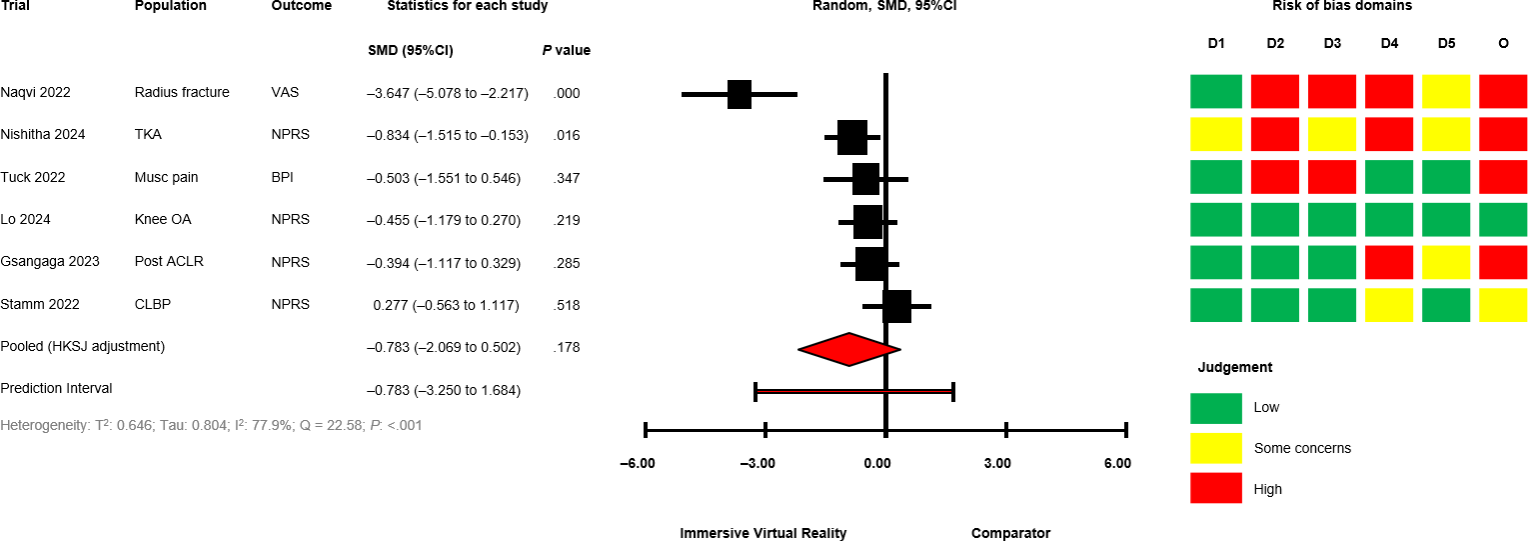
Between-group random effects meta-analysis of pain intensity assessments for trials with an exercising comparator group [29,55,57,58,62,66]. ACLR: Anterior Cruciate Ligament Reconstruction; BPI: Brief Pain Inventory; CLBP: Chronic Lower Back Pain; D1: Domain 1; D2: Domain 2; D3: Domain 3; D4: Domain 4; D5: Domain 5; HKSJ: Hartung-Knapp-Sidik-Jonkman; Musc: Musculoskeletal; NPRS: Numerical Pain Rating Scale; O: Overall; OA: Osteoarthritis; SMD: standardized mean difference; TKA: Total Knee Arthroplasty; VAS: Visual Analog Scale.

**Figure 7.**
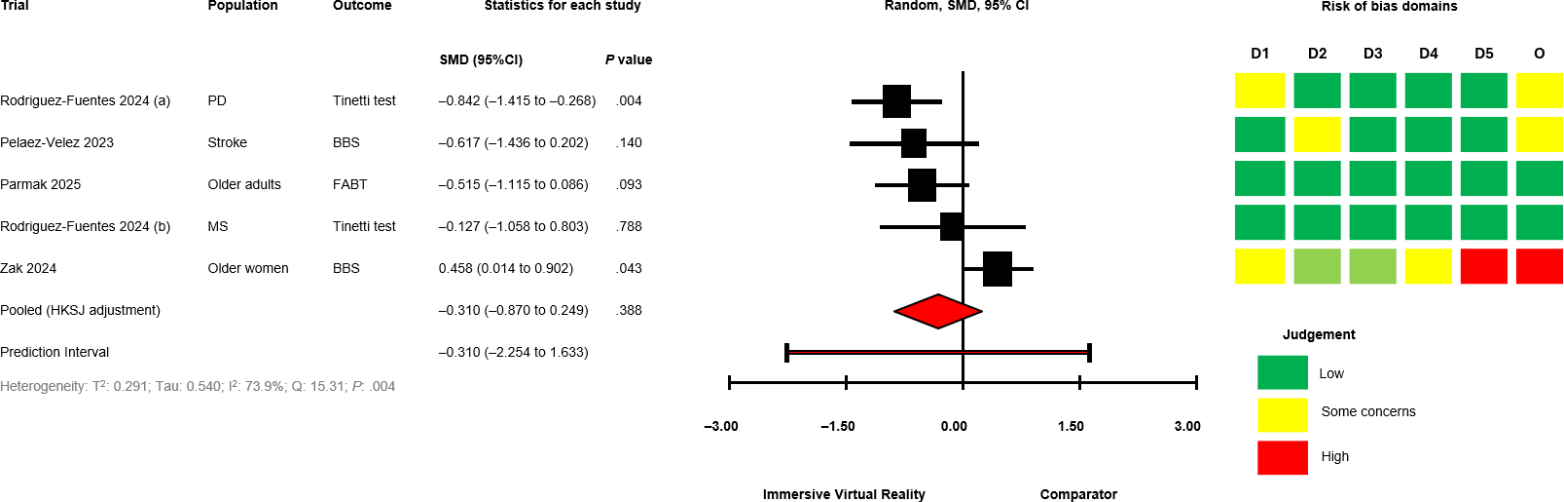
Between-group random effects meta-analysis of composite static and dynamic balance assessments for trials with an exercising comparator group [49-51,68,69]. BBS: Berg Balance Scale; D1: Domain 1; D2: Domain 2; D3: Domain 3; D4: Domain 4; D5: Domain 5; FABT: Fullerton Advanced Balance Scale; HKSJ: Hartung-Knapp-Sidik-Jonkman; MS: Multiple Sclerosis; O: Overall; PD: Parkinson disease; SMD: standardized mean difference.

**Figure 8.**
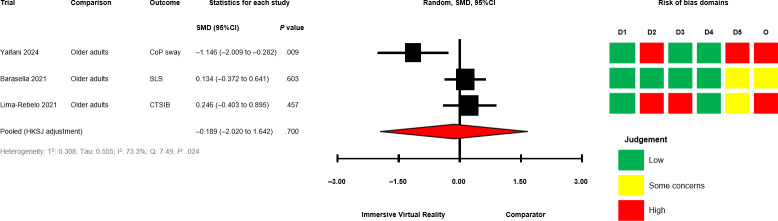
Between-group random effects meta-analysis of static balance assessments for trials with a nonexercising comparator group [60,64,67]. CoP: Center of Pressure; CTSIB: Clinical Test of Sensory Interaction and Balance Test; D1: Domain 1; D2: Domain 2; D3: Domain 3; D4: Domain 4; D5: Domain 5; HKSJ: Hartung-Knapp-Sidik-Jonkman; O: Overall; SLS: Single Leg Stand test; SMD: standardized mean difference.

**Figure 9.**
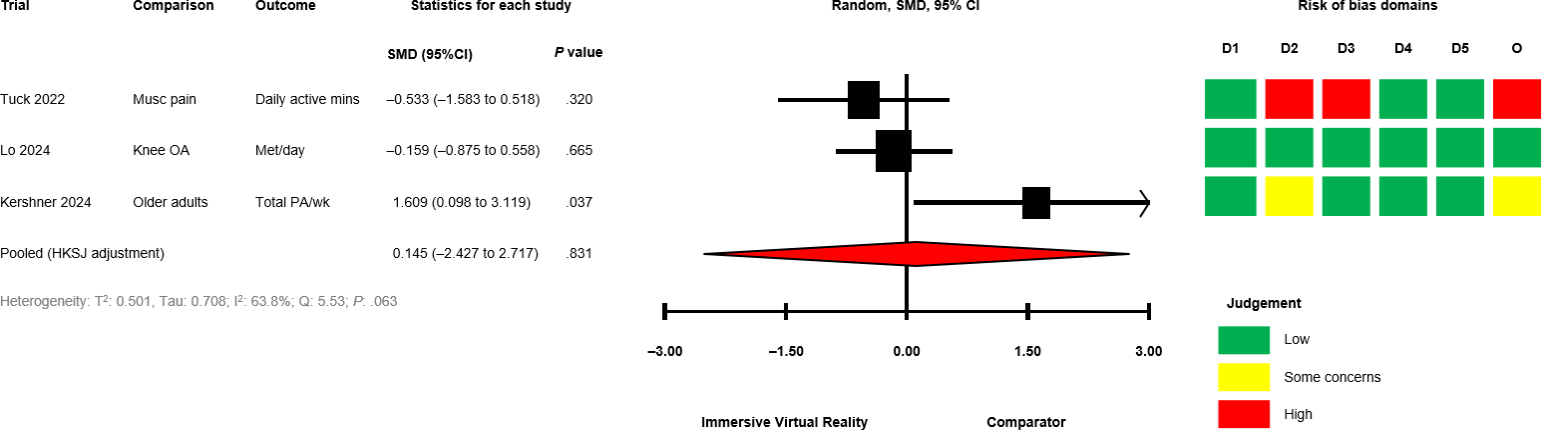
Between-group random effects meta-analysis of aerobic physical activity (device-measured) assessments for trials with an exercising comparator group [29,63,66]. D1: Domain 1; D2: Domain 2; D3: Domain 3; D4: Domain 4; D5: Domain 5; HKSJ: Hartung-Knapp-Sidik-Jonkman; Met: Metabolic Equivalent; Musc: Musculoskeletal; O: Overall; OA: Osteoarthritis; PA: Physical Activity; SMD: standardized mean difference.

The between-group analyses for IVR versus comparator, grouped by comparator group activity (exercising/nonexercising) are presented in Figure 2 [28,50,51,58,60,64,67-71] for mobility and functional balance (exercising: SMD −0.345, 95% CI −1.095 to 0.406; *P*=.29; nonexercising: SMD −0.322, 95% CI −0.931 to 0.288; *P*=.22), Figure 3 [28,50,51,56,60,67,69-71] for functional leg strength (exercising: SMD −0.161, 95% CI −0.573 to 0.250; *P*=.34; nonexercising: SMD −0.351, 95% CI −1.750 to 1.049; *P*=.48), Figure 4 [28,29,50,53-55,65,69] for quality of life (exercising: SMD 0.036, 95% CI −0.444 to 0.516; *P*=.85; nonexercising: SMD −0.053, 95% CI −0.839 to 0.728; *P*=.80), Figure 5 [29,49,50,55,57,58,62] for condition severity (SMD −0.153, 95% CI −2.227 to 1.921; *P*=.86), Figure 6 [29,55,57,58,62,66] for pain intensity (SMD −0.783, 95% CI −2.069 to 0.502; *P*=.18), Figure 7 [49-51,68,69] for composite static and dynamic balance assessments (SMD −0.310, 95% CI −0.870 to 0.249; P=.39), Figure 8 [60,64,67] for static balance (SMD −0.189, 95% CI −2.020 to 1.642; *P*=.70) and Figure 9 [29,63,66] for aerobic physical activity (device-measured; SMD 0.145, 95% CI −2.427 to 2.717; *P*=.83).

No significant effects were observed compared with exercising and nonexercising comparators for any outcome domain. Substantial heterogeneity was identified among most exercising (mobility and functional balance: T^2^=0.399, Tau=0.630, *I*^2^=80.3%, Q=25.38, *P*<.01; condition severity: T^2^=2.182, Tau=1.477, *I*^2^=92.7%, Q=82.06, *P*<.01; pain intensity: T^2^=0.646, Tau=0.804, *I*^2^=77.9%, Q=22.58, *P*<.01; composite static and dynamic balance: T^2^=0.291, Tau=0.540, *I*^2^=73.9%, Q=15.31, *P*<.01) and nonexercising (functional leg strength: T^2^=0.471, Tau=0.687, *I*^2^=75.9%, Q=12.46, *P*<.01; static balance: T^2^=0.308, Tau=0.555, *I*^2^=73.3%, Q=7.49, *P*=.02) comparator meta-analyses. Statistical heterogeneity was not identified for functional leg strength (T^2^=0.00, Tau=0.00, *I*^2^=0.0%, Q=2.65, *P*=.61), quality of life (T^2^=0.026, Tau=0.162, *I*^2^=18.4%, Q=4.91, *P*=.30) or aerobic physical activity (device-measured; T^2^=0.501, Tau=0.708, *I*^2^=63.8%, Q=5.53, *P*=.06) exercising meta-analyses, and mobility and functional balance (T^2^=0.096, Tau=0.031, *I*^2^: 42.0%, Q=6.90, *P*=.22) and quality of life (T^2^=0.000, Tau=0.000, *I*^2^=0.0%, Q=0.69, *P*=.71) nonexercising meta-analysis. Calculated prediction intervals were often substantially wider than the 95% CI, indicating significant heterogeneity for most outcome domains (Figures 2-9). Visual inspection of funnel plots did not suggest the presence of small-study effects for all outcome domains (Figures S1-S8 in Multimedia Appendix 1). However, the Egger statistic indicated evidence for a small-study effect for mobility and functional balance outcomes (Figure 2; *P*=.01). Sensitivity analyses identified single study removal of Zak et al [68] (functional balance, composite static and dynamic balance), Nishitha et al [58] (condition severity), Naqvi et al [57] (pain intensity), Stamm et al [55] (pain intensity), and Barsasella et al [60] (functional leg strength) resulted in altered statistical significance in meta-analytical models. Manipulation of pre-post correlation values did not result in altered statistical significance for any meta-analysis.

### Primary Clinical Effectiveness Outcome Domains

#### Overview

The following section is a narrative description of outcomes under each of the 8 primary clinical effectiveness domains. These include outcomes that were unable to be included in meta-analyses due to a lack of pooled comparative data (ie, less than 3 comparable control groups across trials; Table 3, Figures S9-S16 in Multimedia Appendix 1).

#### Mobility and Functional Balance

Mobility and functional balance assessments were reported in 12 trials (46% of total) [28,50-52,58,60,64,67-71]. Between-group differences favoring IVR (*P*<.05) were reported in 4 trials (3 with exercising comparators) [50,58,67,71]. One trial reported a between-group difference favoring an exercising comparator group [68]. Between-group differences were either not statistically significant or unreported in 7 trials [28,51,52,60,64,69,70]. Within-group improvements (*P*<.05) for IVR groups were reported in 5 trials [51,58,60,64,71], and comparator interventions in 4 trials (3 exercising comparators) [52,60,68,71]. One trial reported a within-group deterioration in a nonexercising comparator group [28].

#### Condition Severity

Assessments of change in individual condition severity were reported in 8 trials (31% of total) [29,49,50,52,55,57,58,62]. Between-group differences favoring IVR (*P*<.05) were reported in 3 trials (3 with exercising comparators) [57,58,62]. Between-group differences were either not statistically significant or unreported in 5 trials [29,49,50,52,55]. Within-group improvements (*P*<.05) for IVR were reported in 4 trials [49,57,58,62], and exercising comparators in 2 trials [57,62].

#### Quality of Life

Assessments of change in participant quality of life were reported in 8 trials (31% of total) [28,29,50,53-55,65,69]. Between-group differences favoring IVR (*P*<.05) were reported in one trial with a nonexercising comparator [28], and one with an exercising comparator [50]. Between-group differences were either not significant or unreported in 6 trials [29,53-55,65,69]. Within-group improvement (*P*<.05) for a virtual reality group was reported in one trial [55] and for the mental composite score of the Short Form-12 Health Survey for an exercising comparator [55]. One trial reported a deterioration of the environmental subscale of the World Health Organization Quality of Life Scale questionnaire in an exercising comparator [65].

#### Functional Leg Strength

Measurements of functional leg strength were reported in 9 trials (35% of total) [28,50,51,56,60,67,69-71]. Between-group differences (*P*<.05) favoring IVR were reported in 4 trials (2 with exercising comparators) [28,67,69,71]. Between-group differences were either not significant or unreported in 5 trials (3 exercising comparators) [50,51,56,60,70]. Within-group improvements (*P*<.05) for virtual reality groups were reported in 3 trials [51,56,67,71], and 2 exercising comparators [56,71]. One trial reported significant within-group deterioration in a nonexercising comparator [28].

#### Pain Intensity

Pain intensity outcomes were reported in 6 trials (23% of total) [29,55,57,58,62,66]. Between-group differences (*P*<.05) favoring IVR were reported in 3 trials with exercising comparators [57,58,62]. Between-group differences were either not significant or unreported in 3 trials with exercising comparators [29,55,66]. Within-group improvements (*P*<.05) for IVR groups were reported in 2 trials and their exercising comparators [57,62].

#### Composite Static and Dynamic Balance Assessments

Composite static and dynamic balance assessments were described in 6 trials (23% of total) [28,49-51,68,69]. Three trials reported between-group differences (*P*<.05) favoring IVR (2 with exercising comparators) [28,50,69]. One trial reported a between-group difference favoring an exercising comparator [68]. Between-group differences were either not significant or unreported in 2 trials with exercising comparators [49,51]. Within-group improvements for IVR groups were reported in 4 trials [28,49,50,68], and one exercising comparator [73].

#### Aerobic Physical Activity (Device-Measured)

Physical activity was assessed using device-based measures in 3 trials (12% of total) [29,63,66]. No trials reported any between- or within-group differences (*P*<.05) for any measure.

#### Static Balance

Static balance outcomes were reported in 4 trials (15% of total) [60,64,67,68]. A between-group difference (*P*<.05) favoring IVR was reported for one trial with a nonexercising comparator [67]. Within-group improvement (*P*<.05) favoring IVR was reported in 3 trials [64,67,68], and 2 comparators (one exercising) [64,68].

### Secondary Feasibility Outcome Measures

Feasibility outcomes are reported in Table 4.

**Table 4.** Session attendance, exercise adherence, technological issues, safety, simulator sickness, and participant satisfaction for included trials.

Reference	Session attendance	Exercise adherence	Technological issues (IVR^b^)	Motion sickness (IVR)	Safety	Participant experiences (IVR)
Older adults						
Barsasella et al, 2021 [60]	I^c^ & C^d^: NR^e^	I & C: NR	NR	Simulator sickness description No SS^f^ reported	Adverse events I & C: None observed	NR
Campo-Prieto et al, 2022 [28]	I & C: NR	Percentage of participants completing all sessions I: 92.3% completed all sessions C: NR	NR	SSQ^g^ No SS reported due to VR	Adverse events I & C: None observed	SUS^h^ (total score) SUS: 73.96 (SD 16.77), GEQ PE (domain score) 2.87 (SD 0.86), GEQ NE (domain score) 0.22 (SD 0.21), GEQ T (domain score) 0.37 (SD 0.43), GEQ RR (domain score) 0.19 (SD 0.33), Ad hoc questionnaire Generally positive responses regarding IVR
Drazich et al, 2023 [61]	Mean sessions attended I: 15/16 C: NA	I & C: NR	NR	SSQ No SS reported due to IVR	I & C: NR	AIM^n^: Acceptability (% of participants) 100% found the intervention acceptable; 90% found the intervention highly acceptable AIM^o^: Appropriateness (% of participants) 100% found the intervention appropriate; 70% found the intervention highly appropriate
Kershner et al, 2024 [63]	Total coaching sessions attended I: 59/60 (98%) C: 46/48 (96%)	IVR sessions completed I: 5.5 [0.5]^a^ C: NA	NR	VRSQ^p^ (total score) 2.0 [3.0^a^] (slight or no SS) 44% indicated “no feelings of sickness or discomfort”	Adverse events I & C: None observed	Enjoyment scale (−5 to +5) 5.0 [.0]^a^ Participant survey responses Positive responses in the intervention group for IVR enjoyment and immersiveness
Kwan et al, 2021 [52]	Attendance rate for completers I & C: 100%	NR	NR	VRSQ (total score) 4.63 [18.33^a^]	Study withdrawals I: 1 (experienced mild SS – VRSQ 18.3/100) C: 2 (moderate pain in lower limbs)	NR
Lima Rêbelo et al, 2021 [64]	I & C: NR	Sessions completed I: 14.2 (SD 2.3) 89% C: 14.5 (SD 2.1) 91%	NR	NR	Serious adverse events I & C: None observed	NR
Parmak et al, 2025 [69]	Participation rate I & C: 100%	I & C: NR	NR	VRSQ (total score) 0.09 (SD 0.29)	Adverse events I & C: None observed	VAS satisfaction, I: 9.32 (SD 1.09) C: 7.45 (SD 1.54)
Vorwerg-Gall et al, 2024 [56]	I & C: NR	I & C: NR	NR	NR	I & C: NR	NR
Yalfani et al, 2024 [67]	I & C: NR	I & C: NR	NR	NR	I & C: NR	NR
Zak et al, 2024 [68]	I & C: NR	I & C: NR	NR	NR	Adverse events I & C: None observed	NR
Neurological						
An and Park, 2022 [71]	I & C: NR	I & C: NR	NR	NR	I & C: NR	NR
Peláez-Vélez et al, 2023 [49]	I & C: NR	I & C: NR	NR	NR	I & C: NR	NR
Ramos et al, 2025 [70]	Sessions attended I: 92.7% C: NA	I: NR C: NA	NR	NR	Adverse events I: None observed C: NA^m^	NR
Rodriguez-Fuentes et al, 2024 (a) [50]	Average sessions attended I & C: 42 [range 40‐48 (whole sample)]	I & C: NR	NR	SSQ (Nausea) 7% of intervention group SSQ (Oculomotor) 2.93% of intervention group SSQ (Disorientation) 6.29% of intervention group	Adverse events I & C: None observed	SUS (total score) 82.9/100 GEQ PE^i^ (domain score) 2.92/4 GEQ NE^j^ (domain score) .04/4 GEQ T^k^ (domain score) .23/4 GEQ RR^l^ (domain score) .15/4
Rodriguez-Fuentes et al, 2024 (b) [51]	I & C: NR	Program adherence I: 100% C: NR	NR	SSQ (total score) 1.37/48 (range: 0‐6)	Adverse events I: None observed C: NR	SUS (total score) 90.31/100 (range 72.5‐100)
Vishnuram et al, 2024 [59]	NR	NR	NR	NR	I & C: NR	NR
Musculoskeletal						
Gsangaya et al, 2023 [62]	Session attendance I: 100% C: 100%	Program adherence I: 100% C: 100%	NR	NR	Adverse events I & C: None observed	NR
Lo et al, 2024 [29]	NR	Exercise adherence I: 77.2% [36.8‐104%] C: 62.1% [40.4‐166.0%]	Participant qualitative interviews Qualitative themes show technological challenges and inconvenience of HMD^q^ during exercise	Number of participants reporting SS after exercise 5 Participant qualitative interviews 2 (13%) of participants reported SS throughout the intervention General themes of SS	Adverse events I & C: None observed	Participant qualitative interviews Qualitative outcomes indicate a mix of positive and negative experiences regarding satisfaction and acceptability using the HMD for exercise
Naqvi et al, 2022 [57]	NR	NR	NR	NR	I & C: NR	NR
Nishitha et al, 2024 [58]	NR	NR	NR	NR	I & C: NR	NR
Stamm et al, 2022 [55]	NR	Training units completed, I: 9.18 (SD 1.47) (77%), C: 10.81 (SD 1.6) (90%)	Observed technical issues Connection issues with base stations and internet	NR	Serious adverse events I & C: None observed	Qualitative description of UEQ^r^ IVR was rated as at least “above average” on each individual subscale. “Excellent” rating for attractiveness and perspicuity. “Good” rating for efficiency, dependability, and stimulation “Above average” for originality
Tuck et al, 2022 [66]	NR	Session completion I: All participants completed ≥7 sessions. 30% (3/10) completed 12. C & TG: NR	NR	NR	Adverse events I: None observed C, TG: NR	Participant qualitative interviews Generally positive attitudes towards VR for enjoyment and perceived benefits
Cardiopulmonary						
Rutkowski et al, 2022 [65]	NR	NR	NR	NR	NR	NR
Wang et al, 2023 [53]	NR	NR	NR	NR	MACE^s^ incidence I: 11.11% (1x angina, 1 x arrhythmia) C: 16.67% (1x arrhythmia, 2 x angina, 1 x restenosis)	NR
Cancer						
Schrempf et al, 2023 [54]	NR	Sessions performed as planned I: 75.6% C: NR	NR	NR	Adverse events I: None observed C: NR	EORTC^t^ (total score) I: 72.6
Metabolic						
Seo et al, 2023 [72]	NR	NR	NR	NR	NR	”Exercise fun” 4-point Likert scale I: 32.82 (SD 4.61/40)

a Median [IQR].

b IVR: immersive virtual reality.

c I: intervention.

d C: control.

e NR: not reported.

f SS: simulator sickness.

g SSQ: simulator sickness questionnaire.

h SUS: system usability scale.

i GEQ PE: game experience questionnaire - positive experience.

j GEQ NE: game experience questionnaire - negative experience.

k GEQ T: game experience questionnaire - tiredness.

l GEQ RR: game experience questionnaire - returning to reality.

m NA: not applicable.

n AIM: acceptability of intervention measure

o AIM: appropriateness of intervention measure.

p VRSQ: virtual reality sickness questionnaire.

q HMD: head-mounted display.

r UEQ: user experience questionnaire.

s MACE: major adverse cardiovascular event.

t EORTC: European Organization for Research and Treatment of Cancer inpatient satisfaction 32-item questionnaire.

#### Safety Metrics

Safety metrics were reported in 16 trials (62% of included studies) [28,29,50-55,60,62-64,66,68-70]. Fifteen studies reported adverse events incidence [28,29,50,51,53-55,60,62-64,66,68-70], and one trial reported safety-related study withdrawals for the IVR and exercising comparator groups [52]. No study-related adverse events were reported. No trials reported an increased number of adverse events in the IVR groups compared with comparators. Fourteen trials (88% of trials reporting safety metrics) reported no adverse events in any study group [28,29,50,51,54,55,60,62-64,66,68-70].

#### Session Attendance/Exercise Adherence

Session attendance was reported in 7 trials (27% of total) [50,52,61-63,69,70]. For trials differentiating between groups, attendance was 98% for IVR exercise sessions and 99% for exercising comparators [52,61-63,69]. Adherence to exercise prescription was reported in 9 trials (35% of total). For trials providing a calculable percentage of session completion by participants, IVR groups completed an average 87% of sessions (n=7) [28,29,51,54,55,62,64], and comparator interventions completed 94% (n=3) [55,62,64].

#### Motion Sickness

Motion sickness in response to IVR exposure was reported in 9 trials (32% of total) [28,29,50-52,60,61,63,69]. Generally, low levels of motion sickness were reported after IVR exercise interventions via quantitative assessments. One trial described low levels of motion sickness through participant qualitative interviews [29]. One trial terminated the intervention for one participant due to persistent motion sickness symptoms [29].

#### Technical Issues

Two trials (8% of total) [29,55] reported technical issues for IVR interventions. Common technical issues reported were primarily due to disturbance to the Internet connection and HMD inconvenience.

#### Participant Experiences

Eleven trials (42% of total) [28,29,50,51,54,55,61,63,66,69,72] reported measures related to participant experiences with IVR exercise post intervention. For qualitative assessments, responses generally indicated positive user experiences and high usability for IVR, with participants citing overall enjoyment, motivation, and beneficial perceived benefits [29,66]. Negative experiences with IVR primarily surrounded the discomfort and inconvenience of using an HMD during exercise sessions [29].

### Risk of Bias in Trials and Confidence in Cumulative Outcomes

Risk of bias assessments for individual trials progressing to meta-analyses are presented alongside forest plots in (Figures 2-9). Risk of bias proportions for separate outcome domains are presented in Table S2 in Multimedia Appendix 1. Many trials exhibited issues with measurement and selection of reported results across all outcome domains (domains 4 and 5 on the Cochrane RoB2 tool). In turn, 75% of outcomes included across all meta-analyses were rated as “some concerns” (n=18) or at “high” risk of overall bias (n=21). GRADE assessments for outcome domains progressing to meta-analysis are presented in Table S2 in Multimedia Appendix 1. All outcome domains were deemed to have “low” or “very low” certainty levels. Risk of bias, indirectness of intervention, and imprecision of effects had the main impacts on the level of certainty.

## Discussion

### Principal Findings

This systematic review with meta-analyses assessed the clinical effectiveness of IVR interventions using aerobic or anaerobic exercise. The analysis combined 26 RCTs, including 846 participants. Population groups included adults with neurological disorders, older adults, musculoskeletal conditions, cardiopulmonary diseases, metabolic conditions, and cancer. Pooled data indicated no differences compared with the comparator for clinical effectiveness, with high levels of statistical and clinical heterogeneity and low certainty ratings leading to decreased confidence in findings. While scarcely reported among included trials, findings were generally positive regarding the feasibility of IVR exercise. This has important ramifications for both practitioners who are seeking to adopt IVR as a novel alternative for exercise delivery and researchers conducting future trials.

### Clinical Effectiveness of Immersive Virtual Reality Exercise Interventions

For all clinical effectiveness outcome domains progressing to meta-analysis, certainty in effect was classified as “very low” or “low.” Many included trials exhibited issues with the measurement and selection of reported results, leading to increased risk of bias. Limited comparative data, small sample sizes, substantial heterogeneity, and increased risk of bias across all outcomes decreased certainty in all findings. This outlines a need for robust clinical trials to be conducted to improve confidence in observed findings.

Pooled data seem to suggest that aerobic or anaerobic exercise via IVR may be comparable to exercising controls. This observation seems to be common across supervised and unsupervised comparator groups across populations. Recent evidence suggests that IVR may be effective for mitigating short-term pain intensity during and post exercise (≈30% reduction) [74], although evidence on longer-term pain is unclear [75]. Attentional distraction and altered pain modulation may be potential mechanisms enhanced in IVR [76]. This review identified a nonsignificant effect favoring IVR for pain intensity compared with exercising controls, marred by high statistical and clinical heterogeneity across population groups. The relationship between IVR exercise and pain intensity outcomes should be emphasized in future robust clinical trials.

No significant differences were observed between IVR and nonexercising comparators for all meta-analysis outcome domains. This observation may be in part due to a low number of trials with nonexercising comparators progressing to meta-analyses. This may have led to a potential lack of power to detect any differences in the meta-analytical models. Exercise prescription, supplementary equipment, and additional intervention components received by the IVR groups varied substantially across all trials. While it cannot be expected that all weekly physical activity be completed in IVR, most included trials either did not meet the World Health Organization physical activity [77] or American College of Sports Medicine resistance exercise guidelines [78] (inclusive of other intervention elements) or did not report exercise prescription in sufficient detail. This indicates a potential issue with appropriate intervention dosage and a lack of reporting for activities being completed outside of the trial context. This lack of clarity in dosage or reporting could have contributed to the nonimprovement of relevant outcomes and may help to partly explain meta-analysis findings.

Inclusion criteria for this review mandated the inclusion of aerobic or anaerobic exercise as part of the IVR intervention. As such, it is surprising that common exercise-related outcome measures were not regularly assessed (eg, muscular fitness and cardiorespiratory fitness). A 2024 systematic review identified that IVR improved physical activity enjoyment, intrinsic motivation, and exercise intention compared with traditional and nonimmersive physical activity interventions [20]. Muscular and cardiorespiratory fitness outcomes were not often assessed across trials. Given the clinical importance of these outcomes for overall health [79,80], future research should prioritize their collection. A 2022 RCT in apparently healthy adults used a resistance training-based IVR intervention compared with a self-directed exercise approach [24]. The trial found that IVR exercise improved pertinent cardiometabolic and physical fitness measures (eg, muscular strength, systolic blood pressure) significantly more than control, possibly due to gamification elements improving exercise adherence [24]. Future research should assess whether resistance training-based IVR has a similar effect on cardiometabolic outcomes for clinical populations. Given the broad population inclusion criteria in the current review, there was a lack of literature from certain chronic condition populations where exercise interventions may be used for disease control (eg, type 2 diabetes) [81]. In addition to the populations identified in this review, future work should attempt to assess clinical effectiveness outcomes in these groups.

### Feasibility of IVR Exercise Interventions

Determining the feasibility of IVR exercise for service delivery is important for widespread implementation. Safety concerns for IVR exercise may be compounded by technology-related factors (eg, immersiveness decreasing spatial awareness) contributing to potential injury risk [82]. However, regular physical activity and exercise are often critical for condition management [83-85], and IVR presents a unique opportunity to stimulate exercise enjoyment [20]. Previous literature suggests that IVR is safe for physical rehabilitation [86-88], but exercise-specific literature is lacking. While IVR may present an opportunity to deliver exercise in a novel way, individual risk analysis is imperative to address safety concerns. This is particularly true for population groups susceptible to IVR-specific side effects, such as cognitive overload and motion sickness [89]. While only just over half of the trials reported safety metrics (14/26, 56%), no trials in the current review reported an increased exercise-related adverse event rate in IVR groups for any population.

A common side effect reported from IVR interventions is motion sickness, commonly derived from eye strain, disorientation, or nausea [90]. Recent literature reports high variability concerning the incidence of motion sickness, with between 25% and 60% of participants experiencing symptoms [91]. Occurrence of motion sickness in IVR is multifaceted and linked to hardware type (eg, display type and mode, time delay), IVR content (eg, duration, controllability, realism), and individual susceptibility [90,91]. This review identified minimal-slight severity symptoms, indicating that motion sickness was not common or severe in controlled environments. However, findings regarding motion sickness were not commonly reported among trials (8/25, 32%). This is surprising given the high incidence of motion sickness symptoms in IVR and the unclear additional effects of exercise. Therefore, for clinicians using IVR exercise, we recommend an individualized approach to combat potential motion sickness symptoms. This includes (1) selecting IVR hardware and content based on patient feedback [90,91], (2) accounting for motion sickness history and IVR-readiness [90,91], and (3) using IVR interventions based on safety guidelines for individual systems [92].

Perceived technical issues have been cited as a barrier to the implementation of IVR among health care professionals [93]. This includes technical malfunctioning of IVR equipment, unstable Internet connectivity, and system usability [93]. Technical issues were poorly reported in the current review, with only 2 trials describing experiences with technical challenges. Given that a perceived lack of experience/confidence and fear of technical issues disrupting clinical sessions may dissuade clinicians from using IVR [93], future trials should prioritize their reporting and troubleshooting techniques. While trials in the current review suggest that attendance to sessions and adherence to exercise prescription were high, they were not reported commonly and lacked standardized presentation. The inability to verify whether participants attended and completed prescribed sessions makes it difficult to determine whether the IVR exercise was associated with the observed findings. This notion is supported by a 2024 systematic review of physiotherapist-led telerehabilitation interventions, which identified a lack of standardized reporting for attendance and adherence [94]. Since these are often linked with clinical effectiveness, more nuanced and comprehensive investigations into attendance and adherence are warranted [94].

Generally positive participant experiences were observed for participants using the IVR exercise in the included trials. Self-report questionnaires identified high levels of enjoyment and motivation, system usability, and overall acceptability among participants. Qualitative descriptions of experience found that perceived personal benefits and novelty of IVR may be the driving factors for positive experiences. These themes are consistent throughout the literature, with high degrees of immersion [32], distraction [32,95], and incorporation of gamification (eg, leaderboards) [32,96] often cited as factors influencing positive experiences with IVR. Sources of negative experiences related to the inconvenience of wearing an HMD for exercise. A 2022 systematic review observed that older adults reported several problems with wearing an HMD [97]. These included that the HMD was too heavy, caused general discomfort, and resulted in negative affect [97]. Many participants were willing to tolerate these discomforts to experience positive IVR features (such as immersive networking) [97]. These findings, combined with the current review, may suggest that the selection of IVR exercise should be individualized and based on participant preference.

### Future Directions

Due to the advancement of IVR in recent years, the potential for integration into exercise practice is immense. This review identifies that future trials should investigate the clinical effectiveness of IVR exercise with more rigorous methodology. Future studies should focus on the measurement and selection of the reported results being derived from pre-established protocols to minimize bias, measuring common exercise-related outcome measures, sample size calculations to acquire statistical power, and intention-to-treat analysis to increase the validity of results. Future studies should emphasize the reporting of session attendance and exercise adherence, technical issues, and participant experiences to assess overall feasibility.

This systematic review has several important limitations to consider. Cumulatively, there was a considerable amount of bias among included studies resulting from the measurement and selection of reported clinical effectiveness results. In turn, “low” to “very low” certainty in the findings was identified through GRADE analysis. Both comparative (IVR vs alternate intervention) and additive (IVR + usual care vs usual care) intervention designs were included for exercising comparators, limiting the specificity and interpretability of findings. While SMD allowed for the comparison of multiple outcome measures across different scales, variability in SDs between populations may limit generalizability. This may have resulted in confounding and potentially distorted the observed effects in meta-analyses [98,99]. Additionally, small-sample bias may have been present in meta-analyses [73]. High statistical heterogeneity was observed in most meta-analyses, and sensitivity analyses revealed statistically significant differences in multiple meta-analytical models through the removal of single trials. These could not be accounted for using meta-regression due to the small number of trials included for each outcome and indicate the fragility of meta-analytical models. Additionally, a confidence distribution approach for the calculation of prediction intervals in meta-analyses with small trial numbers [100] could not be implemented through CMA software. Therefore, the reported prediction intervals may underestimate the uncertainty of the generated effects. A large amount of variety in population groups and participants is present in this review, and subgroup analyses by condition domain were not possible due to sparse outcome data. Therefore, overall findings should be observed with caution when applied to specific populations.

### Conclusions

The findings of this systematic review incorporating meta-analyses provide initial evidence for the clinical effectiveness of IVR exercise interventions. The initial evidence may suggest that IVR exercise interventions do not seem to statistically differ from nonexercising or exercising comparators for changes in clinical effectiveness outcomes. However, significant heterogeneity, high risk of potential bias, and low certainty ratings decrease confidence in the observed findings. While the results indicate that IVR may be a viable option for the delivery of exercise, future trials with more robust methodology for the monitoring and reporting of clinical effectiveness outcomes are needed to verify findings. Importantly, future trials should also emphasize reporting of session attendance and exercise adherence, safety metrics, technical issues, and participant experiences. Implementing these measures will limit the risk of bias observed and provide more accurate insights into the effectiveness and real-world applicability of IVR exercise interventions.

## Supplementary material

10.2196/87542 Multimedia Appendix 1 Online supplementary material.

10.2196/87542 Checklist 1 PRISMA checklist.

## Abbreviations

GRADE: Grading of Recommendations, Assessment, Development and Evaluation
HMD: head-mounted display
IVR: immersive virtual reality
PRISMA: Preferred Reporting Items for Systematic Reviews and Meta-Analysis
PRISMA-S: Preferred Reporting Items for Systematic Reviews and Meta-Analyses–Search
RCT: randomized controlled trial
RoB2: revised Cochrane Risk of Bias 2 tool
SMD: standardized mean difference
