# Supplementary material for: Clinical Effectiveness of Immersive Virtual Reality Exercise Interventions: Systematic Review and Meta-Analysis of Randomized Controlled Trials
Source: J Med Internet Res. 2026 Apr 20;28:e87542. doi: 10.2196/87542 (PMC13105859; doi:10.2196/87542)
Supplement: Multimedia Appendix 1 [file jmir-v28-e87542-s001.pdf]

Clinical effectiveness of immersive virtual reality exercise interventions: a systematic review and meta-analysis

Online Supplementary Material

Contents

Supplementary Material 1: Search Strategies 2

Supplementary Material 2: Clinical Effectiveness Outcomes 5

Supplementary Material 3: Funnel Plots 15

Supplementary Material 4: Overall risk of bias proportions 19

Supplementary Material 5: GRADE assessments 23

Supplementary Material 1: Search Strategies

Databases searched 06/01/2026. No search filters were employed in individual databases.

Pubmed

#	Search string	Results
#1	“Virtual Reality Exposure Therapy”[Mesh] OR “Immersive”[Title/Abstract] AND “virtual realit*”[Title/Abstract] OR “fully immersive”[Title/Abstract] OR “VR”[Title/Abstract] OR “HTC VIVE”[Title/Abstract] OR “Oculus”[Title/Abstract] NOT “wiifit”[Title/Abstract] NOT “kinect”[Title/Abstract] NOT “augmented reality”[Title/Abstract] NOT “AR”[Title/Abstract] NOT “xbox”[Title/Abstract] NOT “nonimmersive”[Title/Abstract] NOT “non-immersive”[Title/Abstract] NOT “vestibular rehabilitation”[Title/Abstract]	20,363
#2	"Physical Therapy Modalities"[Mesh] OR "Physical Therapists"[Mesh] OR "Exercise"[Mesh] OR "Exercise Therapy"[Mesh] OR "Cardiac Rehabilitation"[Mesh] OR "Neurological Rehabilitation"[Mesh] OR "exercise*"[Title/Abstract] OR "physical activit*"[Title/Abstract] OR "strength training"[Title/Abstract] OR "resistance training"[Title/Abstract] OR "aerobic training"[Title/Abstract] OR "endurance training"[Title/Abstract] OR "physiotherap*"[Title/Abstract] OR "physio-therap*"[Title/Abstract] OR "physiatrist"[Title/Abstract] OR "rehabilitation*"[Title/Abstract] OR "physical therap*"[Title/Abstract] OR "physical-therap*"[Title/Abstract] OR "movement"[Title/Abstract] OR "motor"[Title/Abstract] OR “Rehabilitation”[Title/Abstract] OR "tai chi"[Title/Abstract] OR "yoga"[Title/Abstract] OR "balance training"[Title/Abstract] OR "balance retraining"[Title/Abstract] OR "gait training"[Title/Abstract] OR "postural training"[Title/Abstract] OR "strengthening"[Title/Abstract] OR "aerobics"[Title/Abstract] OR “functional training”[Title/Abstract] OR “pilates”[Title/Abstract] OR “stretching”[Title/Abstract] OR “neurological therap*”[Title/Abstract] OR “neurological training” [Title/Abstract] OR “sport*”[Title/Abstract] OR “physical”[Title/Abstract] OR “fitness”[Title/Abstract] OR “cardiometabolic”[Title/Abstract] OR “pain”[Title/Abstract] OR “function*”[Title/Abstract] NOT “vestibular rehabilitation”[Title/Abstract]	7,797,651
#3	"clinical study"[Publication type] OR "clinical trial"[Text word] OR "randomized controlled trial"[Text word] OR "randomised controlled trial"[Text Word] OR “randomised”[Title/Abstract] OR “randomized”[Title/Abstract] OR “a randomized clinical trial”[Title/Abstract] OR “randomised clinical trial”[Title/Abstract] OR “randomized clinical trial”[Title/Abstract]	1,830,300
#4	"Systematic Review"[Publication type] OR "Meta-Analysis"[Publication type] OR "Clinical Trial Protocol"[Publication type] OR “protocol”[Title] OR “a systematic review and meta analysis”[Title/Abstract] OR “systematic review and meta analysis”[Title/Abstract]	544,714
#5	#1 AND #2 AND #3	2,155
#6	#5 NOT #4	1,640

Web of Science (Clarivate)

#	Search string	Results
#1	(((TS=(Immersive)) AND TS=(virtual realit*)) OR TS=(“VR” OR “Virtual Reality Exposure Therapy” OR “HTC VIVE” OR “Oculus” OR “Meta Quest”)) NOT TS=(“wiifit” OR “kinect” OR “augmented reality” OR “AR” OR “xbox” OR “nonimmersive” OR “non-immersive” OR “vestibular rehabilitation”)	58,047

#2	(TS=("exercise*" OR "physical activit*" OR "strength training" OR "resistance training" OR "aerobic training" OR "endurance training" OR "physiotherap*" OR "physio-therap*" OR "physiatrist" OR "rehabilitation*" OR "physical therap*" OR "physical-therap*" OR "motor" OR “tai chi” OR “yoga” OR “balance training” OR "balance retraining" OR "gait training" OR "postural training" OR "strengthening" OR "hydrotherapy" OR "aerobics" OR “functional training” OR “pilates” OR “stretching” OR “neurological therap*” OR “neurological training” OR “sport*” OR "Cardiac Rehab*" OR "Neurological Rehab*” OR “physical activity” OR “physical therap* modalities” OR “exercise therap*” OR “physical” OR “fitness” OR “cardiometabolic” OR “pain” OR “function*”)) NOT TS=(“vestibular rehabilitation”)	14,398,898
#3	ALL="trial" OR “randomised” OR “randomized”	2,234,731
#4	#1 AND #2 AND #3	2,440
#5	TS=“systematic review" OR “meta analysis” OR “meta-analysis” OR "protocol" OR “systematic review and meta analysis” OR “systematic review and meta-analysis” OR “Review” OR “Meeting Abstract”	4,541,141
#6	#4 NOT #5	1,641

Embase (Elsevier)

#	Search string	Results
#1	‘virtual reality’/exp OR ‘virtual reality head mounted display’/exp OR ‘virtual reality system’/exp OR ‘vr interface’:ti,ab,kw OR ‘vr system (virtual reality)’:ti,ab,kw OR ‘virtual reality interface’:ti,ab,kw OR ‘immersive virtual reality’:ti,ab,kw OR ‘virtual reality system’:ti,ab,kw OR ‘htc vive’/exp OR ‘htc vive’ OR ‘oculus’:ti,ab OR ‘meta quest’:ti.ab OR ‘virtual reality exposure therapy’:ti,ab,kw	52,585
#2	'physiotherapy'/exp OR 'exercise'/exp OR 'kinesiotherapy'/exp OR 'heart rehabilitation'/exp OR 'neurorehabilitation'/exp OR 'exercise*':ti,ab,kw OR 'physical activit*':ti,ab,kw OR 'strength training':ti,ab,kw OR 'resistance training':ti,ab,kw OR 'aerobic training':ti,ab,kw OR 'endurance training':ti,ab,kw OR 'physiotherap*':ti,ab,kw OR 'physio-therap*':ti,ab,kw OR 'physiatrist':ti,ab,kw OR 'rehabilitation*':ti,ab,kw OR 'physical therap*':ti,ab,kw OR 'physical-therap*':ti,ab,kw OR 'movement':ti,ab,kw OR 'motor':ti,ab,kw OR ‘tai chi’:ti,ab,kw OR ‘yoga’:ti,ab,kw OR ‘balance training’:ti,ab,kw OR ‘balance retraining’:ti,ab,kw OR ‘gait training’:ti,ab,kw OR ‘postural training’:ti,ab,kw OR ‘hydrotherapy’:ti,ab,kw OR ‘aerobics’:ti,ab,kw OR ‘functional training’:ti,ab,kw OR ‘sport’/exp OR ‘sport’:ti,ab,kw OR ‘sports’:ti,ab,kw OR ‘exercise performance’:ti,ab,kw OR ‘exercise training’:ti,ab,kw OR ‘fitness training’:ti,ab,kw OR ‘fitness workout’:ti,ab,kw OR ‘physical conditioning, human’:ti,ab,kw OR ‘physical effort’:ti,ab,kw OR ‘physical exercise’:ti,ab,kw OR ‘physical work-out’:ti,ab,kw OR ‘physical workout’:ti,ab,kw OR ‘physical activity’/exp OR ‘activity, physical’:ti,ab,kw OR ‘physical activity’:ti,ab,kw ‘pilates’ OR ‘stretching exercise’:ti,ab,kw OR ‘neurological therapy’:ti,ab,kw OR ‘muscle stretching’:ti,ab,kw OR ‘neurological training’:ti,ab,kw OR ‘pain’:ti,ab,kw OR ‘cardiorespiratory fitness’:ti,ab,kw	1,452,154
#3	'clinical trial'/exp AND ‘random*’:ti,ab,kw AND ‘article’:it OR ‘controlled clinical trial’:ti,ab,kw OR ‘randomized controlled trial’:ti,ab,kw	734,946
#4	'systematic review'/exp OR 'systematic review':ti OR 'meta-analysis':ti OR 'meta analysis':ti OR 'protocol':ti OR 'systematic review and meta-analysis’:ti OR 'systematic review and meta analysis’:ti	835,611

#5	#1 AND #2 AND #3	932
#6	#5 NOT #4	849

Cinahl Complete (EBSCOhost)

#	Search string	Results
#1	MH (“Virtual Reality+”) OR MM ("virtual reality" OR "VR" OR "HTC VIVE" OR "oculus" OR "meta quest") OR AB("virtual reality" OR "VR" OR "HTC VIVE" OR "oculus" OR "meta quest") OR TI ("virtual reality" OR "VR" OR "HTC VIVE" OR "oculus" OR "meta quest")	14,933
#2	MH (“Physical Activity” OR "Physical Therapy+" OR “Sports+” OR “Exercise+” OR "Rehabilitation, Cardiac+" OR “Sports”) OR MM ("Rehabilitation, Vocational" OR “Exercise+” OR “physical activity” OR “sports+”) OR TI ("exercise*" OR "physical activit*" OR "strength training" OR "resistance training" OR "aerobic training" OR “Sports” OR "endurance training" OR "physiotherap*" OR "physio-therap*" OR "physiatrist" OR "rehabilitation*" OR "physical therap*" OR "physical-therap*" OR "movement" OR "motor" OR “tai chi” OR “yoga” OR “balance training” OR "balance retraining" OR "gait training" OR "postural training" OR "strengthening" OR "hydrotherapy" OR "aerobics" OR “functional training”) OR AB ("exercise*" OR "physical activit*" OR "strength training" OR "resistance training" OR "aerobic training" OR "endurance training" OR "physiotherap*" OR "physio-therap*" OR "physiatrist" OR "rehabilitation*" OR "physical therap*" OR "physical-therap*" OR "movement" OR "motor" OR “tai chi” OR “yoga” OR “balance training” OR "balance retraining" OR "gait training" OR "postural training" OR "strengthening" OR "hydrotherapy" OR "aerobics" OR “functional training” OR “Sports”)	724,172
#3	PT “clinical trial” OR TX “clinical trial” OR TX “randomized” OR TX “randomised”	750,270
#4	#1 AND #2 AND #3	1,424
#5	PT “systematic review” OR PT “Meta-Analysis” OR PT “Meta Analysis” OR TI “Protocol”	237,633
#6	#4 NOT #5	1,111

Supplementary Material 2: Clinical Effectiveness Outcomes

Table S1: Clinical Effectiveness Outcomes

Reference	Intervention		Control	
	Baseline	Post-intervention	Baseline	Post-intervention
Older adults				
**Barsasella, 2021[17]**	*Functional leg strength: 30s Sit to Stand Test (repetitions) - none*			
	21.6 ± 9.0	22.0 ± 7.8	19.8 ± 7.3	19.8 ± 7.2
	Mobility & functional balance: 8-Foot Up and Go Test (s) - none			
	7.5 ± 2.7	6.5 ± 2.5	7.0 ± 2.7	5.7 ± 1.7
	Static balance: Single Leg Stance (s) - none			
	16.6 ± 9.6	15.8 ± 10.1	14.5 ± 10.4	15.1 ± 9.7
	Body composition: Weight (kg)			
	58.0 ± 8.7	57.7 ± 9.0	60.7 ± 17.1	60.6 ± 17.1
	Body composition: Body Mass Index (kg/m^2)			
	23.4 ± 2.7	23.3 ± 2.8	23.8 ± 5.9	23.7 ± 5.9
	Body composition: Waist Circumference (cm)			
	79.0 ± 8.1	78.3 ± 9.7	83.3 ± 11.2	84.4 ± 13.3
	Body composition: Hip Circumference (cm)			
	94.1 ± 5.5	93.2 ± 5.6	97.2 ± 8.6	97.1 ± 8.6
	Body composition: Waist-Hip Ratio (score)			
	0.8 ± 0.1	0.8 ± 0.1	0.8 ± 0.1	0.9 ± 0.1
	Blood pressure: Systolic Blood Pressure (mmHg)			
	126.6 ± 20.3	125.0 ± 21.6	126.3 ± 15.4	135.1 ± 13.0
	Blood pressure: Diastolic Blood Pressure (mmHg)			
	67.7 ± 11.1	69.9 ± 12.0	73.1 ± 10.9	79.5 ± 13.3
	Neuromuscular strength: Arm Curl Test (repetitions)			
	28.8 ± 9.1	32.6 ± 9.5	25.5 ± 6.3	28.7 ± 11.5
	Estimated cardiorespiratory fitness: Two-Minute Step Test (steps)			
	119.7 ± 28.5	129.5 ± 28.6	109.9 ± 24.2	116.1 ± 21.3

	Flexibility: Chair Sit and Reach Test (cm)			
	2.5 ± 4.0	2.1 ± 4.6	0.5 ± 4.7	1.1 ± 6.2
	Flexibility: Back Scratch Test (cm)			
	-0.4 ± 5.2	-0.4 ± 4.8	-0.005 ± 2.8	-1.5 ± 4.7
	Happiness: Chinese Happiness Inventory (score)			
	1.3 ± 0.3	1.5 ± 0.5	1.3 ± 1.3	1.3 ± 0.4
**Campo-Prieto, 2022[1]**	*Functional leg strength: 5x Sit to Stand Test (s)*			
	15.6 ± 4.5	13.8 ± 3.5	21.2 ± 12.6	25.6 ± 14.2
	Mobility & functional balance: Timed Up and Go Test (s)			
	17.9 ± 6.4	19.0 ± 6.6	23.2 ± 9.3	26.3 ± 11.8
	Quality of life: Short Form-12 Health Survey (SF12): Mental Component Summary (score) - none			
	48.8 ± 8.7	53.4 ± 8.7	50.4 ± 10.5	55.2 ± 9.5
	Quality of life: Short Form-12 Health Survey (SF12): Physical Component Summary (score)			
	48.8 ± 9.5	48.5 ± 9.0	42.1 ± 12.7	44.4 ± 9.9
	Composite static & dynamic balance: Tinetti Test (score)			
	22.4 ± 2.2	25.3 ± 1.7	21.4 ± 3.6	20.5 ± 3.4
	Neuromuscular strength: Hand Grip Strength (kg)			
	15.5 ± 7.1	20.5 ± 6.2	15.0 ± 6.4	17.0 ± 6.0
**Drazich, 2023**	*Self-reported physical activity: Yale Physical Activity Survey (YPAS): Total Time Summary Index (score)*			
	27.9 ± 13.2	29.1 ± 15.6	29.8 ± 18.9	28.7 ± 10.1
	Self-reported physical activity: Yale Physical Activity Survey (YPAS): Energy Expenditure (score)			
	88.0 ± 35.1	89.3 ± 28.6	87.6 ± 54.4	94.3 ± 35.6
	Self-reported physical activity: Yale Physical Activity Survey (YPAS): Activity Dimensions (score)			
	39.3 ± 9.4	60.6 ± 18.2	34.2 ± 24.5	46.1 ± 20.4
	Depression: Patient Health Questionnaire-8 (PHQ-8) (score)			
	3.3 ± 3.5	2.3 ± 2.6	5.8 ± 3.3	4.7 ± 4.5
**Kershner, 2024[20]**	*Device-measured physical activity: Weekly Total Physical Activity (min) - none*			
	773.8 ± 243.0	807.77 ± 189.6	322.0 ± 137.5	705.5 ± 244.1
	Device-measured physical activity: Daily Steps (steps)			
	5578.0 ± 1494.1	6206.7 ± 2520.8	2783.8 ± 913.1	5687.3 ± 3786.3

	Device-measured physical activity: Weekly Moderate-Vigorous Physical Activity (min)			
	125.4 ± 59.5	187.3 ± 97.7	88.6 ± 85.6	179.8 ± 149.6
	Exercise capacity: Six-Minute Walk Test (m)			
	405.5 ± 59.4	477.8 ± 122.3	420.4 ± 90.7	429.0 ± 117.4
**Kwan, 2021**	*Cognition: Montreal Cognitive Assessment (MoCA) (score)*			
	20.0 ± 3.5	24.0 ± 5.0	20.5 ± 4.5	22.5 ± 4.5
	Mobility & functional balance: Timed Up and Go Test (s)			
	14.0 ± 4.2	10.5 ± 4.2	15.5 ± 6.0	10.8 ± 6.1
	Condition severity: Fried Frailty Phenotype (score) - none			
	2.0 ± 1.0	1.0 ± 1.0	2.0 ± 1.8	1.0 ± 1.0
	Neuromuscular strength: Hand Grip Strength (kg)			
	14.7 ± 8.0	15.7 ± 4.7	14.0 ± 4.6	15.3 ± 5.4
**Lima Rêbelo, 2021[21]**	*Mobility & functional balance: Timed Up and Go Test (s)[a]*			
	NR	-1.71 ± 2.3	NR	-1.22 ± 3.6
	Static balance: Clinical Test of Sensory Interaction and Balance C.1 (score)[a]			
	NR	1.0 ± 6.8	NR	1.5 ± 6.9
	Static balance: Clinical Test of Sensory Interaction and Balance C.2 (score)[a]			
	NR	2.6 ± 8.3	NR	4.1 ± 10.6
	Static balance: Clinical Test of Sensory Interaction and Balance C.3 (score)[a]			
	NR	3.9 ± 11.6	NR	7.3 ± 12.1
	Static balance: Clinical Test of Sensory Interaction and Balance C.4 (score)[a]			
	NR	8.0 ± 11.8	NR	13.2 ± 14.6
	Dynamic balance: Dynamic Gait Index (score)[a]			
	NR	3.0 ± 3.6	NR	3.9 ± 3.6
	Dynamic balance: Functional Reach Test (cm)[a]			
	NR	4.3 ± 7.6	NR	8.6 ± 8.1
	Dizziness: Dizziness Handicap Inventory (score)[a]			
	NR	-7.5 ± 11.7	NR	2.0 ± 22.8
**Parmak, 2025**	*Composite static & dynamic balance: Fullerton Advanced Balance Scale (score)*			
	28.8 ± 4.7	34.5 ± 3.8[b]	27.3 ± 6.6	30.2 ± 6.1

	Upper arm muscular endurance: Weightlifting Task (repetitions)			
	14.7 ± 2.2	17.9 ± 1.6[b]	14.6 ± 2.6	14.8 ± 2.0
	Functional leg strength: 30s Sit to Stand test (repetitions)			
	12.1 ± 3.2	13.9 ± 2.4[b]	10.7 ± 2.9	11.8 ± 2.7
	Estimated cardiorespiratory fitness: Two-Minute Step Test (repetitions)			
	59.1 ± 9.8	63.9 ± 10.6	61.6 ± 15.1	63.1 ± 13.3
	Flexibility: Sit and Reach Test (cm)			
	3.6 ± 7.2	4.7 ± 7.0[b]	0.2 ± 5.0	0.5 ± 5.5
	Flexibility: Back Scratch Test (score)			
	-13.0 ± 9.6	-11.8 ± 8.9	-13.7 ± 7.5	-13.1 ± 7.6
	Mobility & functional balance: Eight-Step Walk Test (s)			
	5.9 ± 0.9	5.6 ± 0.9	6.1 ± 1.1	5.7 ± 1.1
	Fatigue: FACIT Fatigue Scale (total score)			
	11.8 ± 9.7	7.5 ± 5.9	12.9 ± 9.0	10.8 ± 6.0
	Quality of Life: World Health Organization Quality of Life Instrument: Older Adults (score)			
	80.3 ± 8.2	83.9 ± 9.8	74.4 ± 10.0	77.6 ± 11.0
**Vorwerg-Gall, 2024[10]**	*Functional leg strength: 5x Sit to Stand Test (s)[a]*			
	NR	-1.0 ± 1.4	NR	-1.5 ± 1.9
	Blood pressure: Systolic Blood Pressure (mmHg)			
	143.3 ± 77.1	133.0 ± 59.9	147.7 ± 41.1	138.3 ± 57.4
	Blood pressure: Diastolic Blood Pressure (mmHg)			
	83.3 ± 43.9	74.3 ± 39.9	81.2 ± 14.4	80.3 ± 33.4
	Body composition: Weight (kg)			
	75.6 ± 14.1	74.8 ± 13.6	76.0 ± 11.9	75.1 ± 11.9
	Body composition: Body Mass Index (kg/m^2)			
	26.8 ± 3.5	26.6 ± 3.5	27.4 ± 3.0	27.1 ± 3.0
	Body composition: Body Fat Percentage (%)			
	31.1 ± 6.9	30.7 ± 7.0	34.3 ± 5.2	33.9 ± 5.2
	Body composition: Visceral Fat (arbitrary unit)			
	9.7 ± 3.2	9.4 ± 3.2	9.6 ± 2.8	9.4 ± 2.8

	Body composition: Skeletal Muscle Percentage (%)			
	42.3 ± 6.3	42.6 ± 6.3	39.1 ± 4.2	39.3 ± 4.2
	Respiratory function: Forced Vital Capacity (FVC (mean value %)			
	83.5 ± 10.3	83.2 ± 11.9	86.8 ± 18.6	85.6 ± 17.6
	Respiratory function: Forced Expiratory Volume in 1 Second (FEV1) (mean value %)			
	84.6 ± 14.7	92.8 ± 13.1	96.0 ± 23.2	95.5 ± 25.0
	Respiratory function: FEV1/FVC ratio (score)			
	80.6 ± 8.1	85.0 ± 6.9	84.3 ± 7.0	84.6 ± 8.7
	Estimated cardiorespiratory fitness: Physical Working Capacity (watts)			
	89.1 ± 34.4	92.4 ± 35.7	81.8 ± 33.7	81.8 ± 27.6
	Physical function: Short Physical Performance Battery (score)			
	11.4 ± 0.7	11.7 ± 0.4	10.7 ± 2.7	11.3 ± 1.7
**Yalfani, 2024[24]**	*Functional leg strength: 30s Sit to Stand Test (repetitions)*			
	10.3 ± 1.8	12.1 ± 1.6	10.1 ± 1.5	9.3 ± 2.1
	Static balance: Centre of Pressure Anteroposterior Sway (N/cm^2)			
	18.5 ± 7.5	11.3 ± 4.3	12.3 ± 2.64	14.3 ± 3.7
	Static balance: Centre of Pressure Mediolateral Sway (N/cm^2)			
	8.9 ± 2.3	6.6 ± 0.5	7.7 ± 2.4	8.3 ± 2.4
	Static balance: Centre of Pressure Velocity Sway (N/cm^2)			
	8.4 ± 2.0	5.5 ± 1.8	8.0 ± 2.4	7.7 ± 2.9
	Mobility & functional balance: Timed Up and Go Test (s)			
	12.6 ± 2.2	9.6 ± 1.6	11.7 ± 2.1	11.3 ± 2.2
**Zak, 2024[25]**	*Mobility & functional balance: Timed Up and Go Test (s)*			
	14.0 ± 1.7	12.6 ± 1.3	13.5 ± 1.1	11.4 ± 1.1
	Composite static & dynamic balance: Berg Balance Scale (score)			
	39.7 ± 1.3	41.9 ± 2.4	39.3 ± 0.9	42.6 ± 2.8
	Static balance: Single Leg Stance (s)			
	8.8 ± 9.0	9.9 ± 8.5	5.4 ± 1.6	7.9 ± 4.9
Neurological				
**An, 2022[11]**	*Functional leg strength: 5x Sit to Stand Test (s)*			

	54.1 ± 6.7	35.2 ± 7.8	52.6 ± 6.5	37.5 ± 10.0
	Mobility & functional balance: Timed Up and Go Test (s)			
	23.2 ± 4.1	14.5 ± 4.4	22.3 ± 3.2	15.3 ± 4.7
	Gait speed: Ten-Meter Walk Test (s)			
	47.3 ± 5.2	33.5 ± 4.7	45.9 ± 5.9	36.4 ± 8.7
**Ramos, 2025**	*Mobility & functional balance: Timed Up and Go Test (s)*			
	8.5 ± 1.9	7.9 ± 1.6	8.7 ± 1.9	8.9 ± 2.3
	Upper arm muscular endurance: 30s Bicep Curl Test: Right Arm (repetitions)			
	12.0 ± 3.0	15.0 ± 3.0	11.0 ± 4.0	12.0 ± 3.0
	Upper arm muscular endurance: 30s Bicep Curl Test: Left Arm (repetitions)			
	13.0 ± 3.0	15.0 ± 4.0	11.0 ± 5.0	13.0 ± 3.0
	Functional leg strength: 30s Sit to Stand Test (repetitions)			
	11.0 ± 3.0	11 0 ± 3.0	9.0 ± 2.0	11.0 ± 3.0
	Central hemodynamics: Augmentation index at 75 beats per minute (%)			
	14.0 ± 15.0	10.0 ± 19.0	17.0 ± 12.0	16.0 ± 21.0
	Central hemodynamics: Augmentation index (%)			
	16.0 ± 12.0	12.0 ± 18.0	19.0 ± 11.0	19.0 ± 20.0
	Central hemodynamics: Augmented Pressure (mmHg)			
	5.0 ± 4.0	5.0 ± 7.0	6.0 ± 4.0	6.0 ± 7.0
	Central hemodynamics: Forward Pressure Wave (mmHg)			
	29.0 ± 4.0	28.0 ± 7.0	29.0 ± 3.0	29.0 ± 3.0
	Central hemodynamics: Backward Pressure Wave (mmHg)			
	15.0 ± 4.0	14.0 ± 4.0	15.0 ± 3.0	15.0 ± 3.0
	Central hemodynamics: Reflection Magnitude (%)			
	49.0 ± 8.0	50.0 ± 10.0	53.0 ± 8.0	54.0 ± 16.0
**Peláez-Vélez, 2023[2]**	*Composite static & dynamic balance: Tinetti Test Gait (score)*			
	5.4 ± 3.0	9.2 ± 2.9	3.6 ± 4.5	5.8 ± 4.0
	Composite static & dynamic balance: Tinetti Test Balance (score)			
	8.6 ± 4.1	13.6 ± 3.1	6.5 ± 6.1	9.1 ± 5.7
	Composite static & dynamic balance: Berg Balance Scale (score)			

	27.0 ± 15.9	46.0 ± 13.1	21.3 ± 22.8	28.9 ± 20.4
	Condition severity: Motricity Index (score)			
	67.1 ± 31.7	84.0 ± 23.1	72.7 ± 37.7	75.7 ± 36.7
	Physical function: Trunk Control Test (score)			
	65.2 ± 23.3	91.6 ± 21.6	46.6 ± 33.6	69.7 ± 29.4
**Rodriguez-Fuentes, 2024 (a)[3]**	*Functional leg strength: 5x Sit to Stand Test (s)*			
	14.3 ± 5.4	13.8 ± 6.7	13.1 ± 3.0	13.2 ± 4.5
	Mobility & functional balance: Timed Up and Go Test (s)			
	14.1 ± 18.3	10.2 ± 6.4	12.5 ± 9.2	13.7 ± 4.5
	Composite static & dynamic balance: Tinetti Test (score)			
	224 ± 4.9	26.4 ± 3.5	25.0 ± 3.9	25.5 ± 3.6
	Quality of life: Parkinson's Disease Questionnaire (score)			
	7.0 ± 5.6	6.4 ± 4.9	10.8 ± 7.5	9.7 ± 6.5
	Condition severity: Movement Disorders Society Modified Unified Parkinson's Disease Rating Scale (score)			
	21.9 ± 18.8	15.1 ± 9.8	42.7 ± 31.3	34.8 ± 23.4
**Rodriguez-Fuentes, 2024 (b)[4]**	*Composite static & dynamic balance: Tinetti Test (score)*			
	26.1 ± 2.1	26.6 ± 1.5	25.2 ± 2.9	25.4 ± 2.3
	Functional leg strength: 5x Sit to Stand Test (s)			
	14.4 ± 4.5	12.8 ± 3.8	12.7 ± 4.7	12.1 ± 2.8
	Mobility & functional balance: Timed Up and Go Test (s)			
	10.7 ± 5.6	9.2 ± 4.7	8.0 ± 1.9	8.7 ± 2.3
	Fatigue: Fatigue Severity Scale (score)			
	38.5 ± 13.4	36.0 ± 15.0	41.2 ± 12.6	39.7 ± 10.9
	Neuromuscular strength: Hand Grip Strength (kg)			
	42.1 ± 9.1	48.5 ± 17.2	43.1 ± 4.3	51.3 ± 5.8
**Vishnuram, 2024**	*Gait speed: Gait Speed (m/s)*			
	0.5 ± 0.0	0.6 ± 0.1	0.4 ± 0.0	0.5 ± 0.1
Musculoskeletal				
**Gsangaga, 2023[19]**	*Pain intensity: Numerical Pain Rating Scale (score)*			
	4.6 ± 0.8	0.4 ± 0.5	4.8 ± 0.9	0.9 ± 0.7

	Condition severity: International Knee Documentation Committee Score (score)			
	82.9 ± 3.2	93.9 ± 1.0	85.0 ± 3.8	92.9 ± 1.2
	Range of motion: Knee flexion: unaffected leg (degrees)			
	123.3 ± 2.4	123.3 ± 2.4	122.7 ± 4.2	122.7 ± 4.2
	Range of motion: Knee flexion: affected leg (degrees)			
	93.3 ± 4.9	112.7 ± 5.9	94.7 ± 5.2	111.3 ± 5.2
	Neuromuscular power: Vertical Jump (cm)			
	63.3 ± 7.0	91.9 ± 1.8	65.9 ± 4.4	90.8 ± 1.9
	Neuromuscular power: Side to Side Jump (jumps)			
	64.0 ± 4.0	92.7 ± 1.7	66.1 ± 4.9	91.9 ± 1.5
	Neuromuscular power: Single Leg Hop (cm)			
	93.1 ± 1.4	94.7 ± 1.2	77.9 ± 5.7	74.9 ± 3.0
**Lo, 2024**[6]	*Pain intensity: Numerical Pain Rating Scale (score) - none*			
	5.9 ± 1.9	4.8 ± 1.8	4.8 ± 1.5	4.6 ± 2.4
	Condition severity: Western Ontario and McMaster Universities Osteoarthritis Index (score) - none			
	902.9 ± 454.8	791.3 ± 425.1	752.0 ± 549.3	742.4 ± 510.5
	Quality of life: EQ-VAS (score) - none			
	62.0 ± 19.6	71.6 ± 12.8	67.3 ± 17.2	67.7 ± 17.4
	Device-measured physical activity: MET/Day (score) - none			
	34.2 ± 1.2	34.4 ± 1.3	35.0 ± 1.4	34.9 ± 2.0
**Naqvi, 2022**[14]	*Pain intensity: Visual Analogue Scale (score)*			
	7.0 ± 0.7	1.77 ± 0.4	7.5 ± 0.5	4.2 ± 0.3
	Condition severity: Disabilities of the Arm, Shoulder and Hand Questionnaire (score)			
	80.2 ± 1.7	13.0 ± 1.7	80.7 ± 2.8	28.1 ± 6.6
	Range of motion: Wrist Extension (degrees)			
	13.3 ± 4.2	63.7 ± 3.9	14.5 ± 4.5	52.7 ± 3.9
	Range of motion: Ulnar Deviation (degrees)			
	11.9 ± 2.7	44.8 ± 3.4	10.9 ± 2.7	35.7 ± 3.9
	Range of motion: Supination (degrees)			
	23.5 ± 7.8	68.7 ± 4.6	22.5 ± 7.5	59.4 ± 1.8

	Neuromuscular strength: Hand Grip Strength (kg)			
	4.4 ± 2.1	48.8 ± 4.3	4.4 ± 2.1	34.6 ± 3.5
**Nishitha, 2024[15]**	*Pain intensity: Numerical Pain Rating Scale (score)*			
	3.4 ± 0.5	1.5 ± 0.5	3.8 ± 0.4	2.3 ± 0.5
	Condition severity: Western Ontario and McMaster Universities Osteoarthritis Index (score)			
	51.2 ± 1.3	14.9 ± 1.0	62.1 ± 1.6	19.0 ± 2.0
	Mobility & functional balance: Timed Up and Go Test (s)			
	23.2 ± 1.6	8.6 ± 1.1	28.7 ± 1.3	12.1 ± 0.9
	Range of motion: Knee Flexion (degrees)			
	62.3 ± 2.5	105.0 ± 4.2	40.3 ± 4.1	94.3 ± 3.1
**Stamm, 2022[9]**	*Pain intensity: Numerical Pain Rating Scale (score)*			
	3.6 ± 2.4	2.9 ± 2.0	2.9 ± 2.4	1.6 ± 1.5
	Condition severity: Hannover Functional Ability Questionnaire (score)			
	73.1 ± 10.6	81.8 ± 11.2	69.8 ± 16.8	72.7 ± 15.7
	Quality of life: Short Form-12 Health Survey (SF12): Mental Component Summary (score)			
	46.4 ± 10.6	48.4 ± 7.1	50.3 ± 7.7	56.2 ± 4.8
	Quality of life: Short Form-12 Health Survey (SF12): Physical Component Summary (score)			
	41.0 ± 7.8	39.3 ± 8.0	35.9 ± 7.9	37.8 ± 7.3
	Kinesiophobia: Tampa Scale for Kinesiophobia (score)			
	19.3 ± 5.9	17.8 ± 4.7	21.6 ± 6.7	20.7 ± 8.1
**Tuck, 2022[23]**	*Pain intensity: Brief Pain Inventory (score)*[*a - none]			
	8.4 ± 1.8	-1.0 ± 0.9	8.1 ± 1.2	-0.2 ± 2.3
	Device-measured physical activity: Daily Active Minutes (mins)[*a - none]			
	NA	19.5 ± 64.5	NA	-21.1 ± 91.5
	Pain interference: Brief Pain Inventory (score)			
	7.5 ± 1.7	-2.1 ± 1.5	7.1 ± 1.5	-1.0 ± 1.4
	Kinesiophobia: Tampa Scale for Kinesiophobia (score)			
	32.3 ± 5.4	-1.6 ± 5.6	34.6 ± 5.4	-4.0 ± 4.6
	Device-measured physical activity: Steps/day (steps)			
	NA	852.0 ± 2934.0	NA	1127.0 ± 2784.0

Cardiopulmonary				
**Rutkowski, 2022**[22]	*Quality of life: World Health Organisation Quality of Life Scale (score)*			
	61.6 ± 16.2	62.8 ± 14.1	59.7 ± 17.9	62.9 ± 16.8
	Exercise capacity: Six-Minute Walk Test (m)			
	502.0 ± 48.4	558.0 ± 76.0	512.0 ± 54.3	552.0 ± 49.1
	Depression & anxiety: Hospital Anxiety and Depression Scale (score)			
	15.5 ± 7.5	10.3 ± 6.5	17.2 ± 9.8	14.6 ± 8.9
**Wang, 2023**[7]	*Quality of life: 36-Item Short Form Survey (score) - none*			
	66.6 ± 12.0	79.7 ± 11.6	66.3 ± 11.5	76.2 ± 11.9
	Exercise capacity: Six-Minute Walk Test (m)			
	352.1 ± 59.4	417.8 ± 41.9	354.2 ± 61.2	455.7 ± 54.4
	Physical function: Short Physical Performance Battery (score)			
	8.8 ± 1.5	11.1 ± 1.2	8.7 ± 1.6	9.8 ± 1.5
	Depression & anxiety: Hospital Anxiety and Depression Scale (score)			
	13.3 ± 3.0	8.5 ± 2.8	13.3 ± 2.5	10.7 ± 2.2
Cancer				
**Schrempf, 2023**[8]	*Quality of life: 5Q-5D-5L (index score) none*			
	0.9 ± 0.1	0.8 ± 0.2	0.9 ± 0.1	0.8 ± 0.2
Metabolic				
**Seo, 2023**	*Body composition: Body Mass Index (kg/m^2)*			
	26.0 ± 2.3	24.7 ± 2.0	25.4 ± 1.7	25.4 ± 1.8
	Depression: Patient Health Questionnaire-8 (score)			
	5.5 ± 4.5	2.5 ± 1.5	6.0 ± 2.8	5.3 ± 3.1

*Exercising control group. [a]Change from baseline.

Supplementary Material 3: Funnel Plots

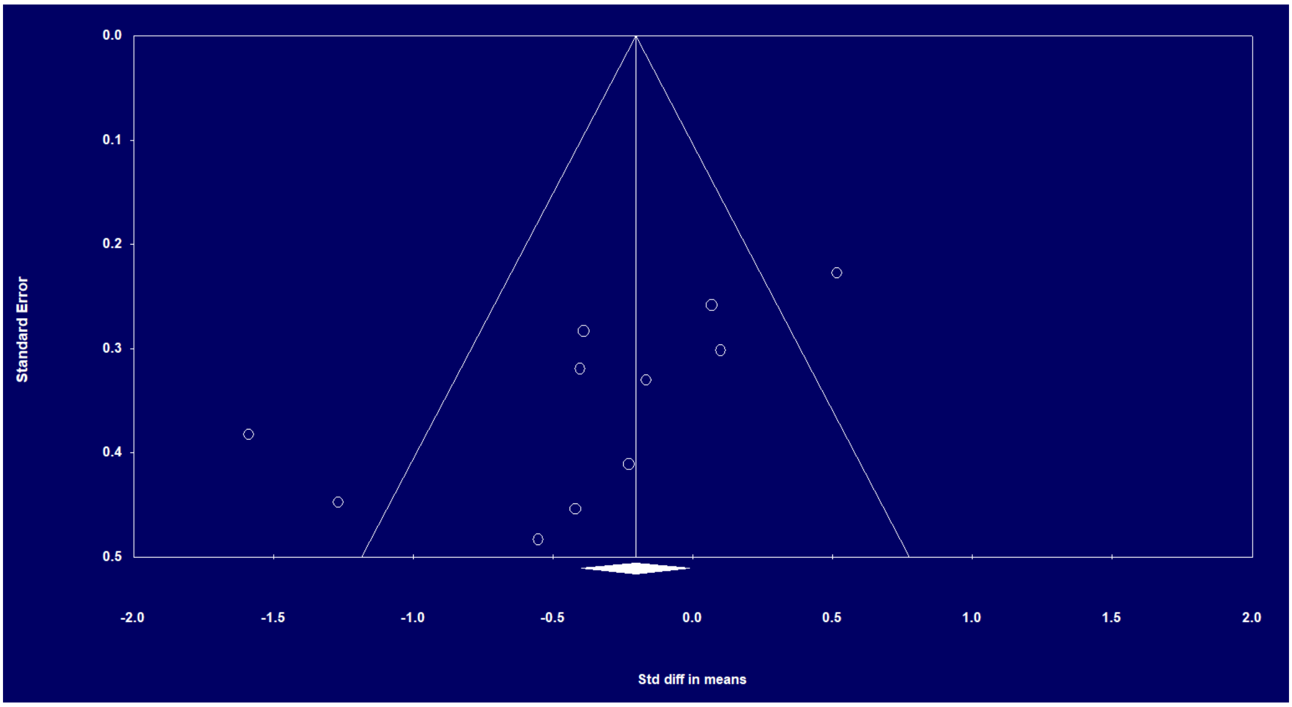

Figure S1 Mobility & functional balance meta-analysis funnel plot

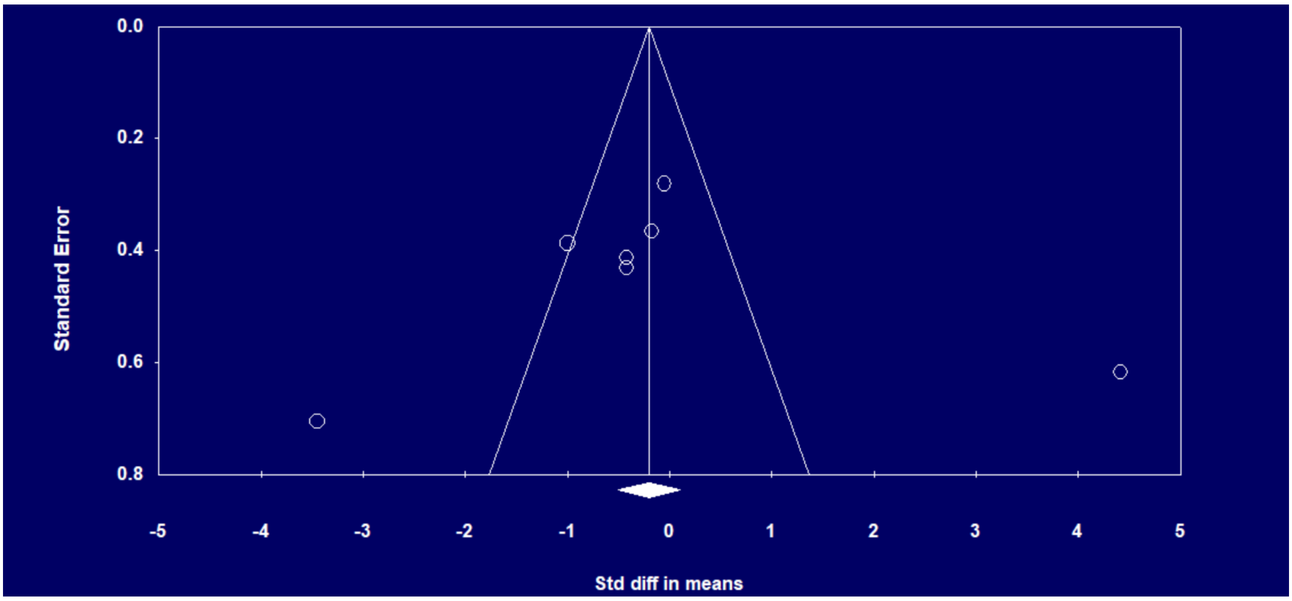

Figure S2 Condition severity meta-analysis funnel plot

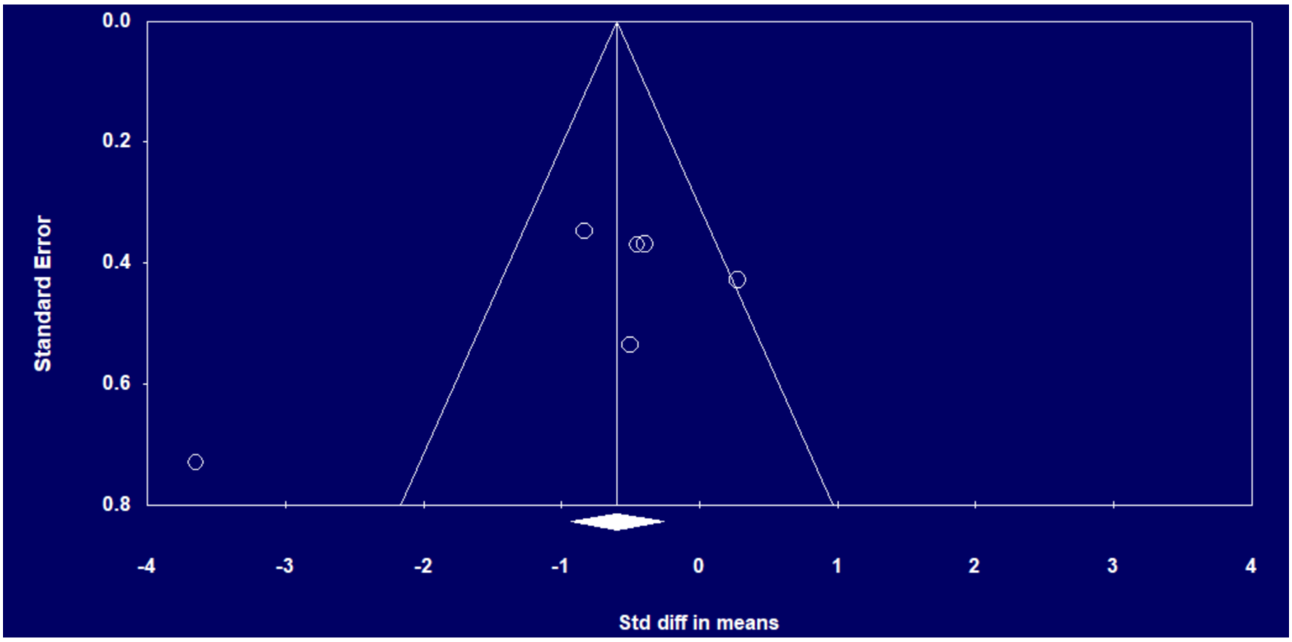

Figure S3 Pain intensity meta-analysis funnel plot

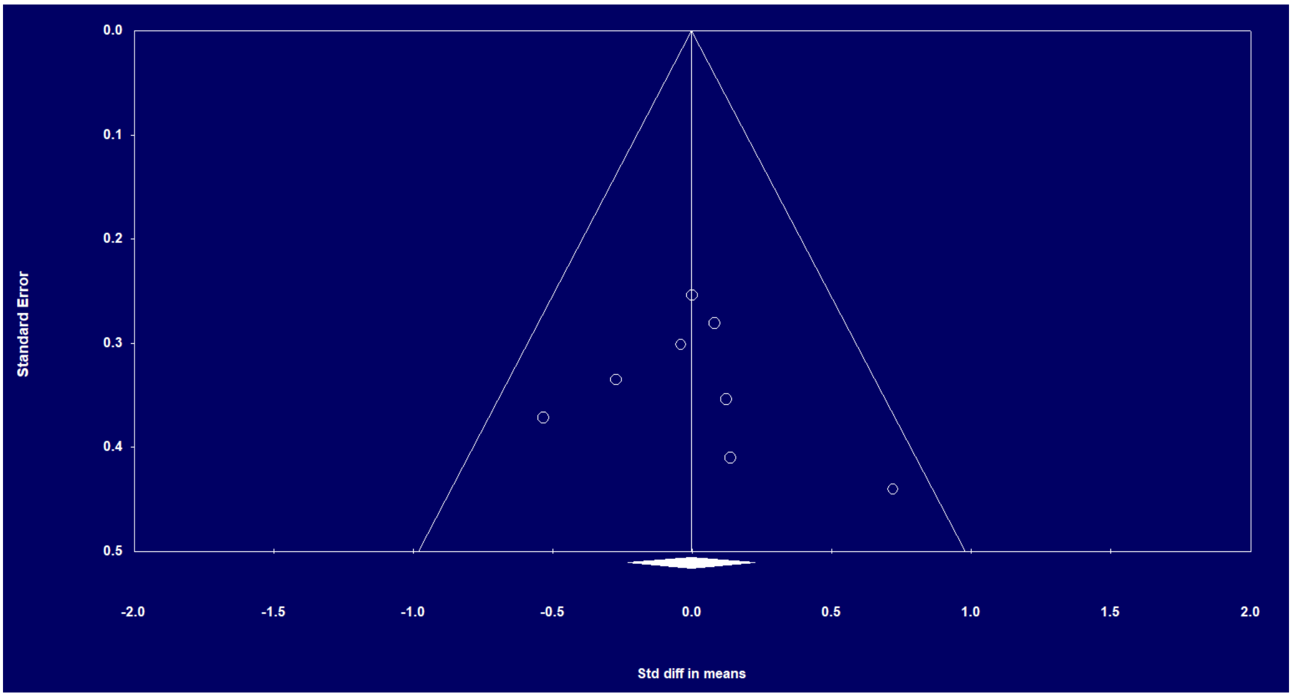

Figure S4 Quality of life meta-analysis funnel plot

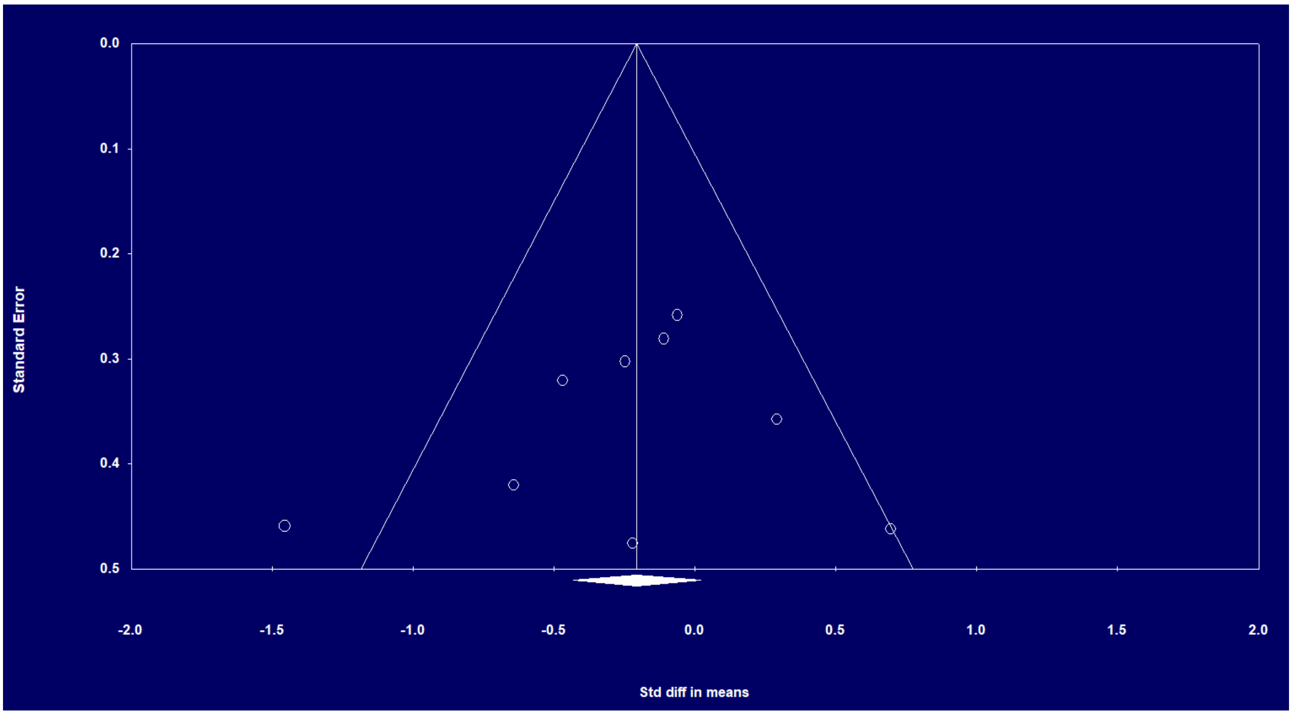

Figure S5 Functional leg strength meta-analysis funnel plot

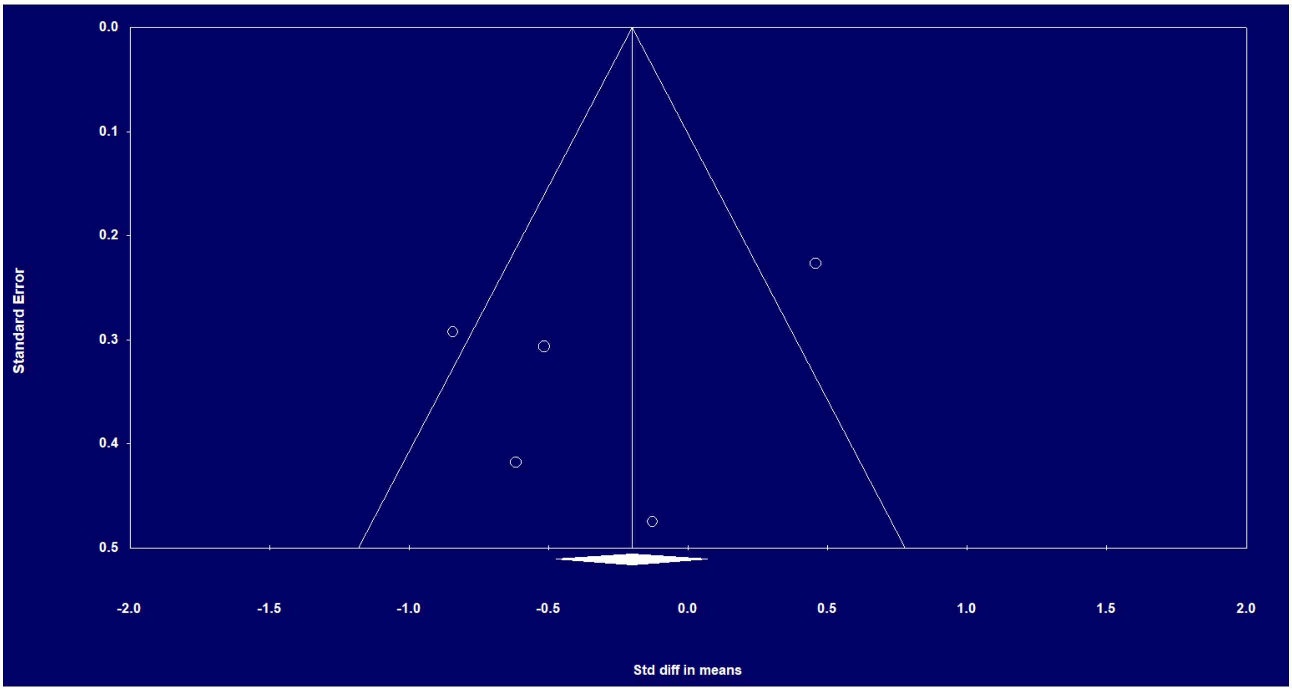

Figure S6 Composite static & dynamic balance assessment meta-analysis funnel plot

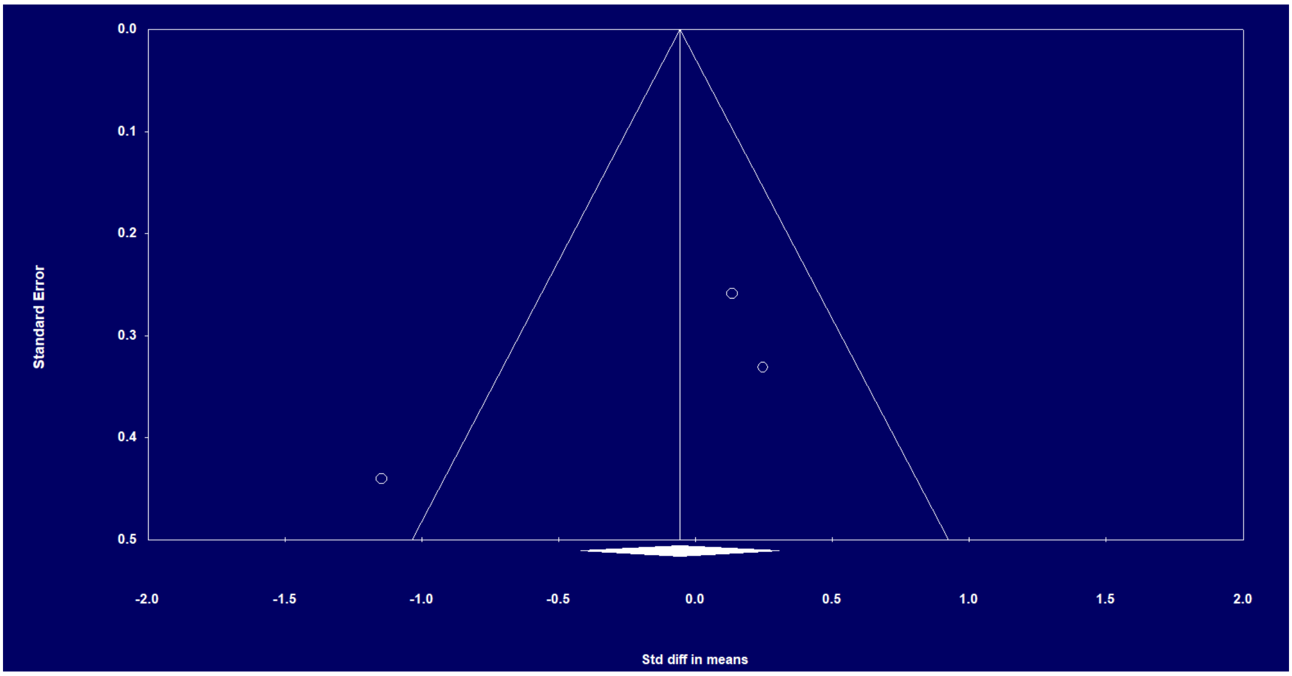

Figure S7 Static balance meta-analysis funnel plot

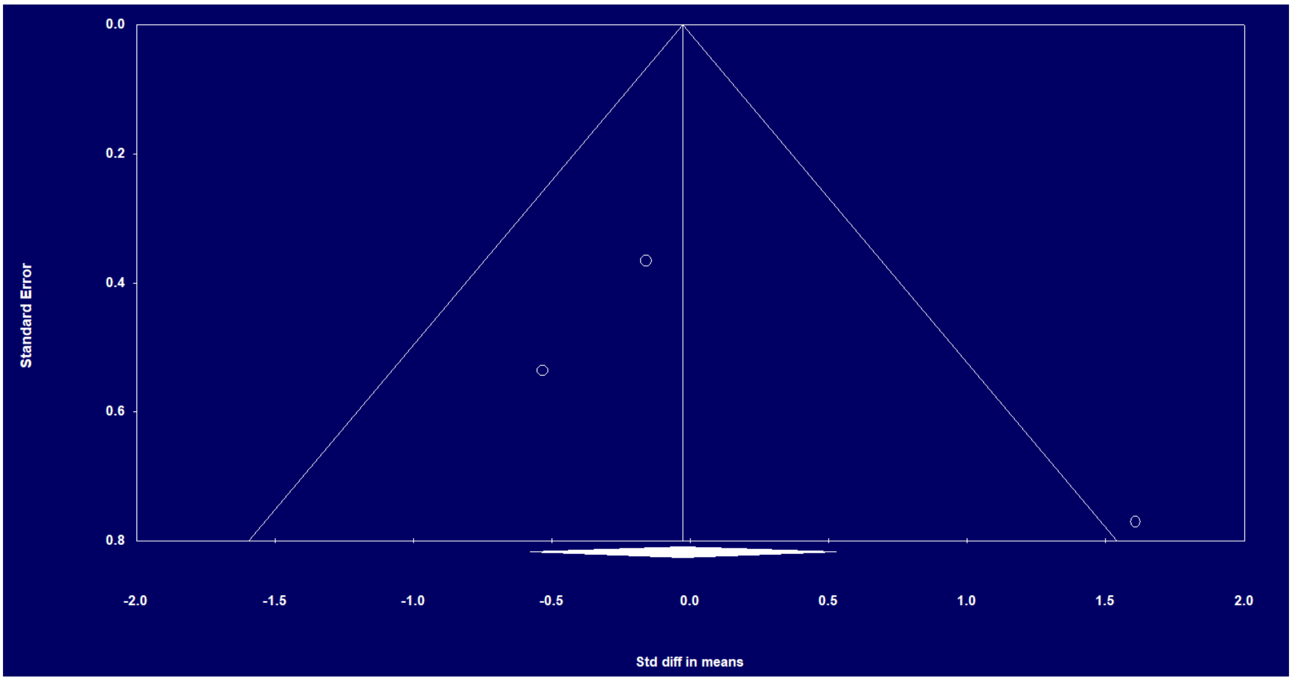

Figure S8 Device-measured physical activity meta-analysis funnel plot

Supplementary Material 4: Overall risk of bias proportions

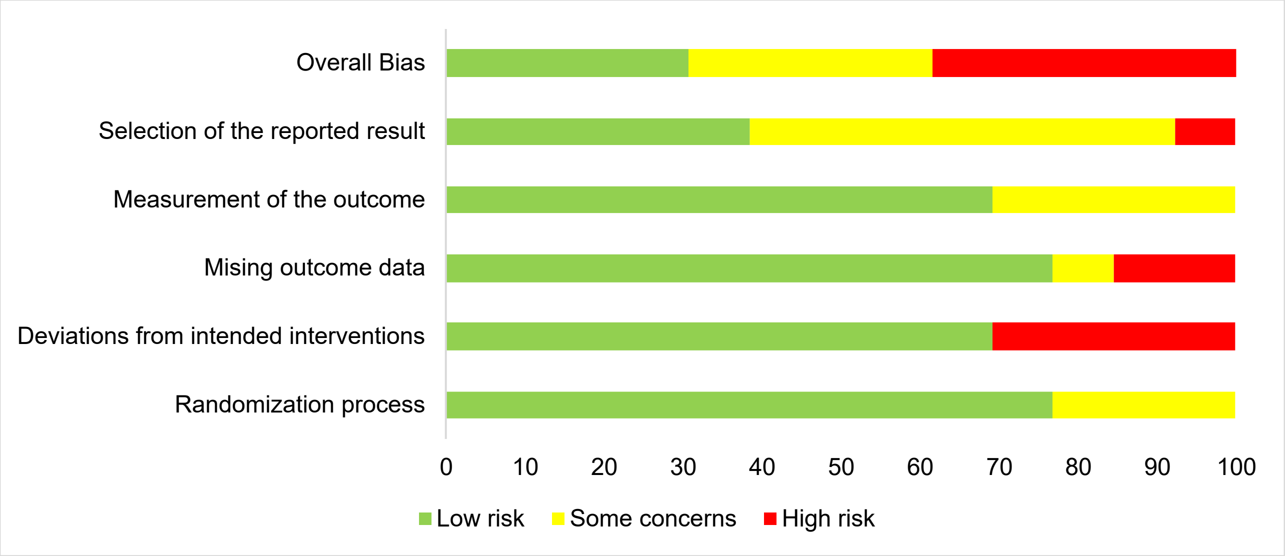

Figure S9 Proportions of risk of bias for mobility & functional balance outcomes

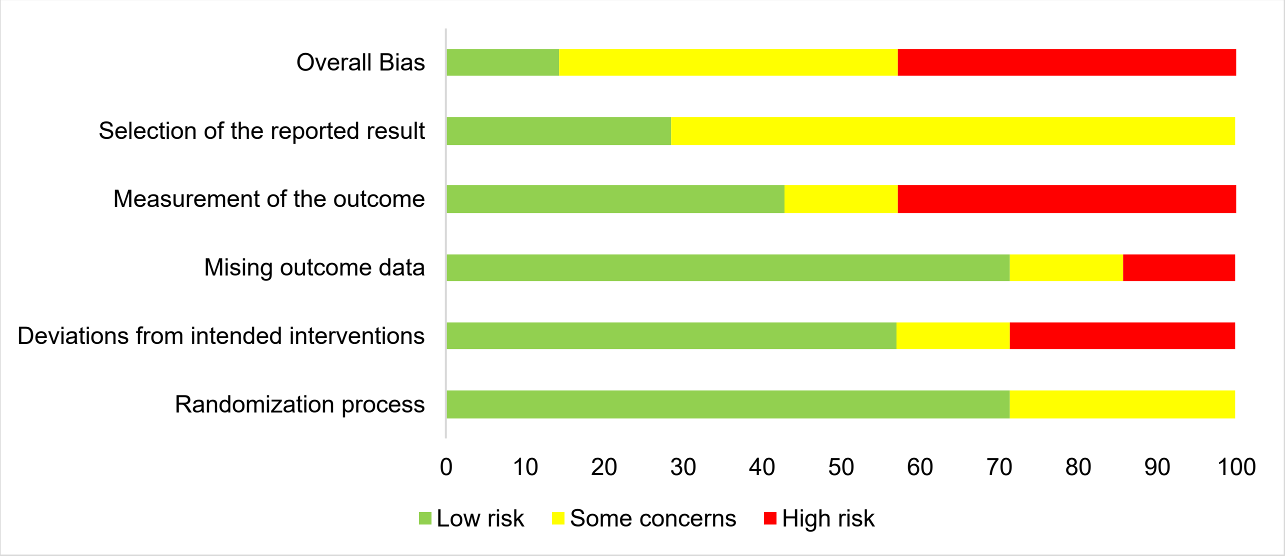

Figure S10 Proportions of risk of bias for condition severity outcomes

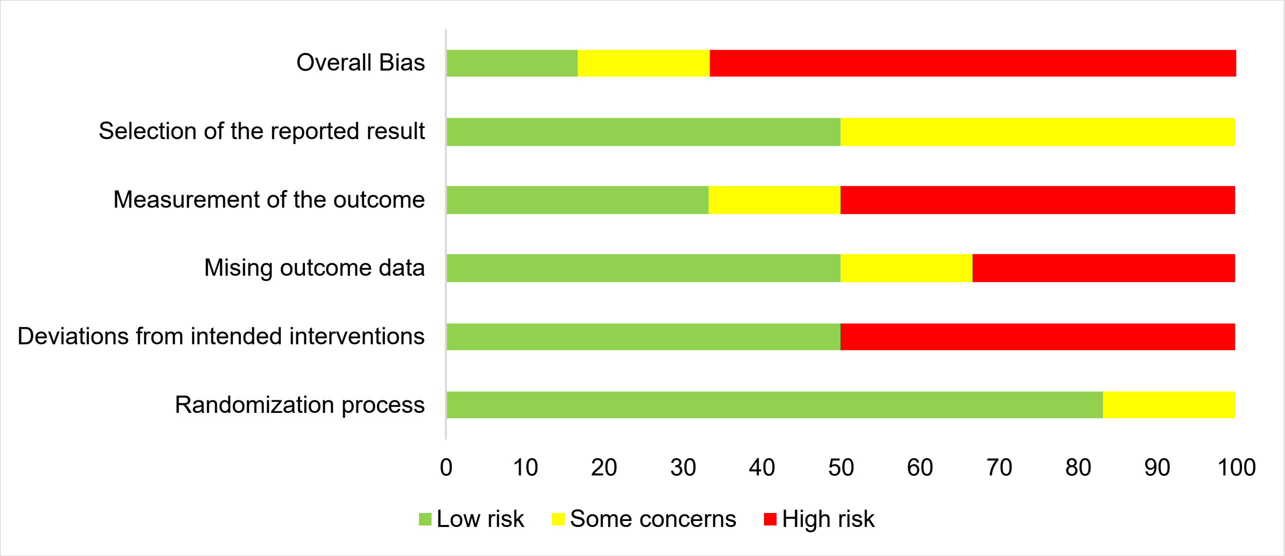

Figure S11 Proportions of risk of bias for pain intensity outcomes

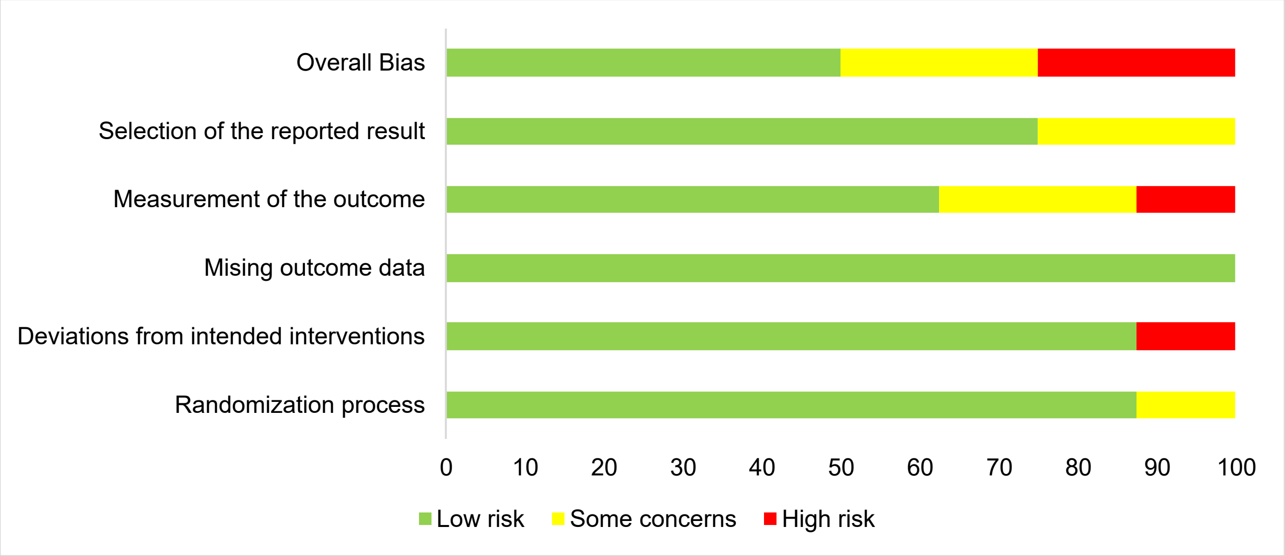

Figure S12 Proportions of risk of bias for quality of life outcomes

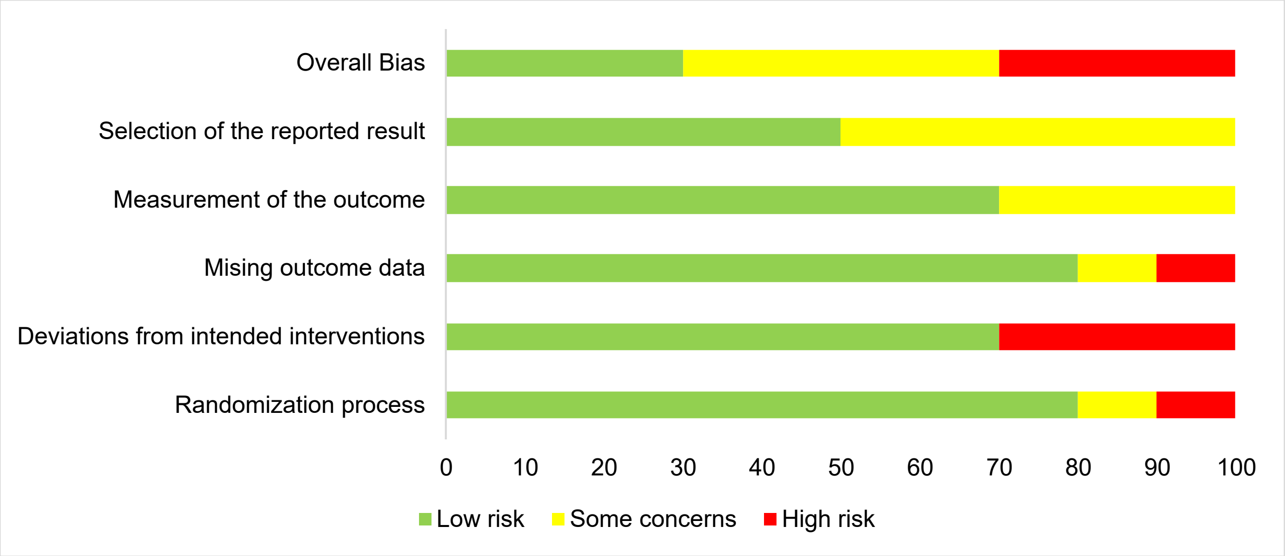

Figure S13 Proportions of risk of bias for functional leg strength outcomes

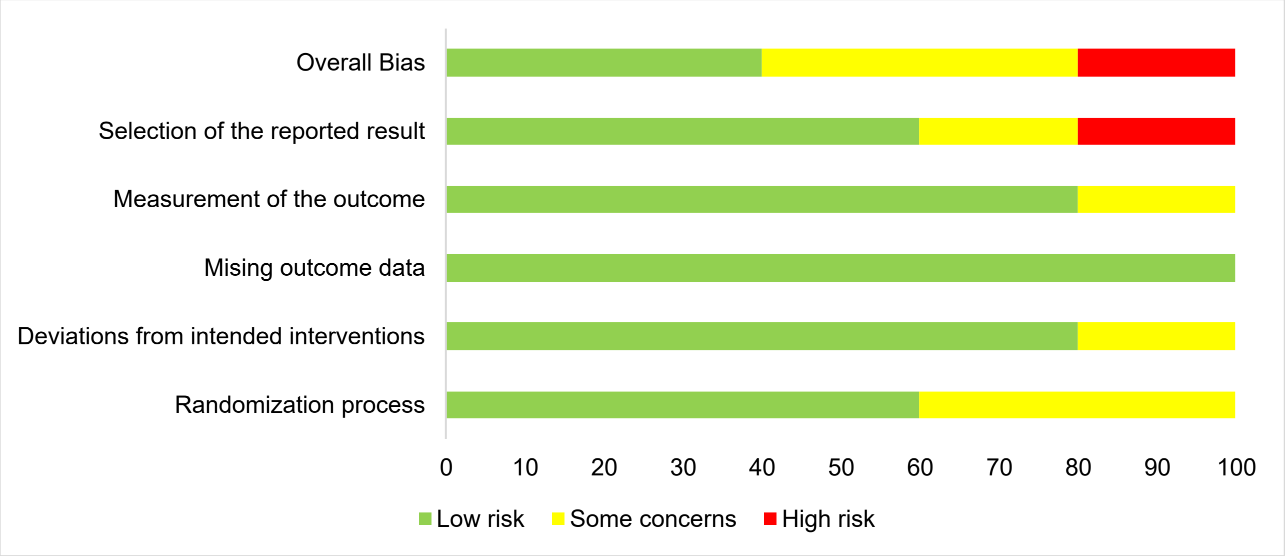

Figure S14 Proportions of risk of bias for composite static & dynamic balance assessment outcomes

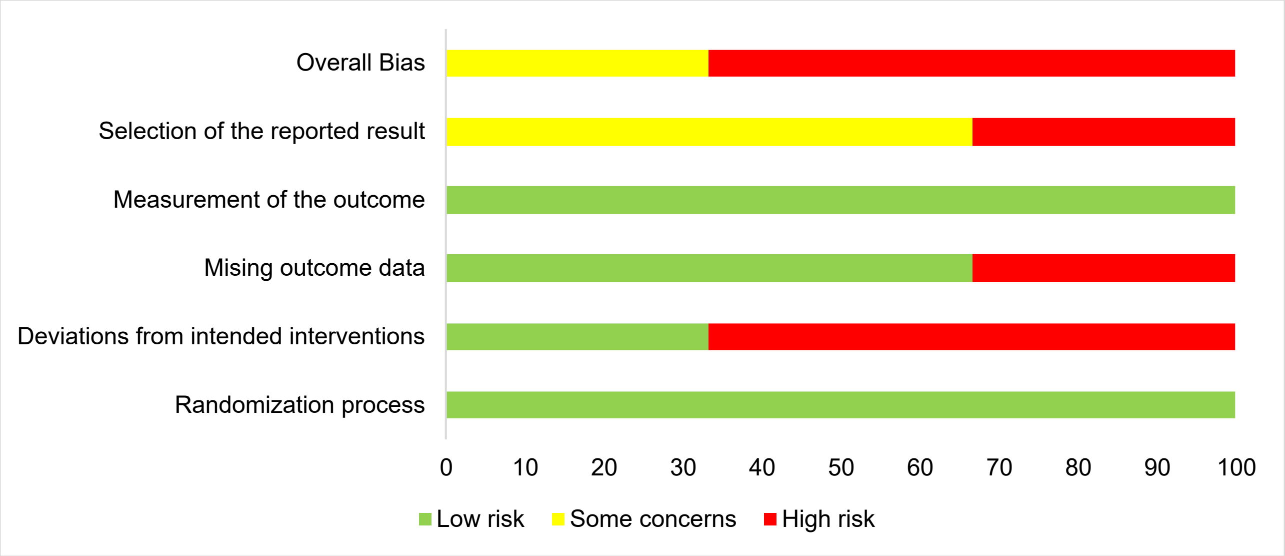

Figure S15 Proportions of risk of bias for static balance outcomes

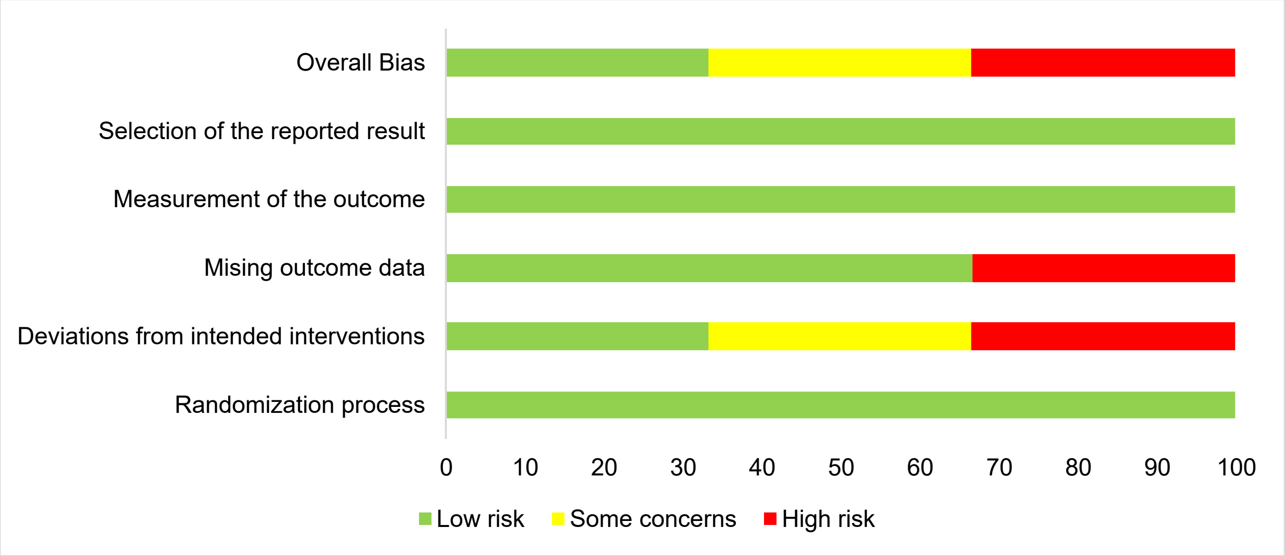

Figure S16 Proportions of risk of bias for device-measured physical activity outcomes

Supplementary Material 5: GRADE assessments

Table S2 GRADE assessments

Certainty assessment							№ of patients		Effect	Certainty	Importance
№ of studies	Study design	Risk of bias	Inconsistency	Indirectness	Imprecision	Other considerations	Immersive Virtual Reality	Comparator	Absolute: SMD (95% CI)		
**Mobility & functional balance:** ***exercising comparator***											
6	Randomised trials	Serious[a]	Serious[b]	Serious[c]	Serious[d]	Publication bias suspected[e]	138	132	**-0.345** (-1.095 to 0.406)	⊕◯◯◯ Very low	Important
**Mobility & functional balance:** ***non-exercising comparator***											
5	Randomised trials	Serious[f]	Not serious	Not serious	Serious[d]	Publication bias suspected[e]	87	79	**-0.322** (-0.931 to 0.288)	⊕◯◯◯ Very low	Important
**Condition severity:** ***exercising comparator***											
7	Randomised trials	Serious[g]	Serious[h]	Serious[i]	Serious[d]	None	111	103	**-0.153** (-2.227 to 1.921)	⊕◯◯◯ Very low	Important
**Pain intensity:** ***exercising comparator***											
6	Randomised trials	Serious[j]	Serious[k]	Serious[l]	Not serious	None	79	79	**-0.783** (-2.069 to 0.502)	⊕◯◯◯ Very low	Important
**Quality of life:** ***exercising comparator***											
5	Randomised trials	Not serious	Not serious	Serious[m]	Serious[d]	None	94	86	**0.036** (-0.444 to 0.516)	⊕⊕◯◯ Low	Important
**Quality of life:** ***non-exercising comparator***											
3	Randomised trials	Serious[n]	Not serious	Not serious	Serious[d]	None	62	60	**-0.053** (-0.839 to 0.728)	⊕⊕◯◯ Low	Important
**Functional leg strength:** ***exercising comparator***											

Certainty assessment							№ of patients		Effect	Certainty	Importance
№ of studies	Study design	Risk of bias	Inconsistency	Indirectness	Imprecision	Other considerations	Immersive Virtual Reality	Comparator	Absolute: SMD (95% CI)		
5	Randomised trials	Serious[o]	Not serious	Not serious	Serious[d]	None	103	77	**-0.161** (-0.573 to 0.250)	⊕⊕◯◯ Low	Important
**Functional leg strength:** ***non-exercising comparator***											
4	Randomised trials	Serious[f]	Not serious	Not serious	Serious[d]	None	67	62	**-0.351** (-1.750 to 1.049)	⊕⊕◯◯ Low	Important
**Composite static & dynamic balance assessments:** ***exercising comparator***											
5	Randomised trials	Serious[o]	Serious[p]	Serious[q]	Serious[d]	None	112	106	**-0.310** (-0.870 to 0.249)	⊕◯◯◯ Very low	Important
**Static balance:** ***non-exercising comparator***											
3	Randomised trials	Serious[f]	Not serious	Not serious	Serious[d]	None	61	60	**-0.189** (-2.020 to 1.642)	⊕⊕◯◯ Low	Important
**Device-measured physical activity:** ***exercising comparator***											
3	Randomised trials	Serious[n]	Serious[b]	Serious[r]	Serious[d]	None	30	29	**0.145** (-2.427 to 2.717)	⊕◯◯◯ Very low	Important

Abbreviations: *CI*, Confidence intervals; *SMD*, standardised mean difference.
Explanations: [a]Four trials (66%) were rated as ‘some concerns’ or higher on RoB2 tool, indicating potential issues with study bias. [b]Variability in meta-analysis plot with CIs not overlapping and large differences with effect magnitude and direction. [c]Three trials (50%) given additional intervention components (e.g. non-IVR exercise program) which may have influenced mobility & functional balance outcomes. [d]Wide CIs including both large effect sizes and no effect. [e]Significant Egger’s statistic, suggestive of potential small study effect (potentially including publication bias). [f]All trials were rated as ‘some concerns’ or higher on RoB2 tool, indicating potential issues with study bias. [g]Six trials (86%) were rated as ‘some concerns’ or higher on RoB2 tool, indicating potential issues with study bias. [h]Variability in the meta-analysis plot with CIs not overlapping and large differences in effect magnitude and direction, significant heterogeneity. [i]Four trials given additional intervention components (e.g. education sessions) that may have influenced condition severity outcomes. [j]Five trials (83%) were rated as ‘some concerns’ or higher on RoB2 tool, indicating potential issues with study bias. [k]Variability in the meta-analysis plot stemming from one trial, significant heterogeneity. [l]Four trials given additional intervention components (e.g. education sessions) that may have influenced pain intensity outcomes. [m]Two trials given additional intervention components (e.g. education sessions) that may have influenced quality of life outcomes. [n]Two trials (67%) were rated as 'some concerns' or higher on RoB2 tool, indicating potential issues with study bias. [o]Three trials (66%) were rated as 'some concerns' or higher on RoB2 tool, indicating potential issues with study bias. [p]Variability in the meta-analysis plot with CIs not overlapping and large differences with effect magnitude and direction, significant heterogeneity. [q]Three trials given additional intervention components (e.g. control intervention in addition) that may have influenced composite static & dynamic balance

assessment outcomes. [r]Two trials given additional intervention components (e.g. behavioural coaching) that may have influenced device-measured physical activity outcomes.
